# Creating a Compassionate World: Addressing the Conflicts Between *Sharing and Caring* Versus *Controlling and Holding* Evolved Strategies

**DOI:** 10.3389/fpsyg.2020.582090

**Published:** 2021-02-10

**Authors:** Paul Gilbert

**Affiliations:** Centre for Compassion Research and Training, College of Health and Social Care Research Centre, University of Derby, Derby, United Kingdom

**Keywords:** compassion, competitive, caring, evolution, strategies

## Abstract

For thousands of years, various spiritual traditions and social activists have appealed to humans to adopt compassionate ways of living to address the suffering of life. Yet, along with our potential for compassion and self-sacrifice, the last few thousand years of wars, slavery, tortures, and holocausts have shown humans can be extraordinarily selfish, callous, vicious, and cruel. While there has been considerable engagement with these issues, particularly in the area of moral psychology and ethics, this paper explores an evolutionary analysis relating to evolved resource-regulation strategies that can be called “*care and share*” versus “*control and hold*.” Control and hold are typical of primates that operate through intimidatory social hierarchies. Care and share are less common in non-human primates, but evolved radically in humans during our hunter-gatherer stage when our ancestors lived in relatively interdependent, small, mobile groups. In these groups, individualistic, self-focus, and self-promoting control and hold strategies (trying to secure and accumulate more than others) were shunned and shamed. These caring and sharing hunter-gatherer lifestyles also created the social contexts for the evolution of new forms of childcare and complex human competencies for language, reasoning, planning, empathy, and self-awareness. As a result of our new ‘intelligence’, our ancestors developed agriculture that reduced mobility, increased group size, resource availability and storage, and resource competition. These re-introduced competing for, rather than sharing of, resources and advantaged those who now pursue (often aggressively) control and hold strategies. Many of our most typical forms of oppressive and anti-compassionate behavior are the result of these strategies. Rather than (just) thinking about *individuals* competing with one another, we can also consider these different resource regulation strategies as *competing within populations* shaping psychophysiological patterns; both wealth and poverty change the brain. One of the challenges to creating a more compassionate society is to find ways to create the social and economic conditions that regulate control and hold strategies and promote care and share. No easy task.

## Overview

All individuals, groups, and societies have to come to terms with the reality of the suffering of life. How we do that has profound implications for the kind of individuals we become and the social communities we create. The basis of our suffering is well known: that all of us have just found ourselves here with a genetically built body and brain we never chose that is vulnerable to disease and injury, lives for a short time before decaying and dying (Dawkins, 1976). Along this journey we will experience the grief of losses of loved ones, conflicts and life setbacks and for some, be in chronic pain and disability. We have minds full of many potential emotions both helpful and harmful (Gilbert, 2018; Nesse, 2019). Our environments make many of us unable to access to clean water, vulnerable to starvation, and diseases that with sufficient resources would be preventable and curable. And one of our (and other animals) greatest sources of suffering is other human beings that go to war, torture and turn other human beings (and animals) into slaves and resources to exploit.

There are three potential responses to suffering that can be referred to as *the big three C’s* (Gilbert, 2005, 2018; Gilbert and Mascaro, 2017): *Compassion* is ‘a sensitivity to suffering in self and others with a commitment to try to alleviate and prevent it’ (Gilbert, 2020a). C*allousness*, is an insensitivity, lack of concern and indifference to suffering sometimes associated with helping being seen as too costly. *Cruelty*, is a deliberate causing of suffering for pleasure or sense of power. This shows up in repressive regimes and our entertainments far more than we would like to admit.

This paper highlights that the evolution of caring behavior, through parent–infant investment, was a template for many forms of caring. Hormones such as oxytocin and vasopressin and a range of physiological changes such as to the autonomic nervous system and frontal cortex evolved with, and tune us into caring behavior (Porges, 2007, 2017; Mayseless, 2016; Carter et al., 2017). There is now considerable evidence that from the day we are born to the day we die the caring and sharing relationships we have with others around us effect our epigenetics, cardiovascular, immune and autonomic nervous systems, and multiple neural circuits underpinning health, prosocial behavior and happiness (for reviews Brown and Brown, 2015, 2017; Ricard, 2015; Seppälä et al., 2017). In addition, caring and sharing became the central social discourse in hunter-gatherer societies as a resource distribution strategy. In this social environment the phenotype for caring possibly became more flexible and variable and extended to the wider social relationships in which individuals matured (Spikins, 2015, 2017). Evidence for this is the fact that most forms of caring and compassionate behavior, be it toward kin, friends or strangers or even animals operate through the same physiological mechanisms (for reviews see Mayseless, 2016; Seppälä et al., 2017). If caring and sharing can be viewed as a phenotype(s) that varies in different contexts, can be linked to a particular resource distribution strategy, and impacts physiological systems, then we should see variations in the psychological and physiological manifestations of this strategy in different environments. To put this another way, some contexts will make us more oriented to caring and sharing and others more orientated to some forms of callousness, and this will show up in physiological as well as psychological processes. The evidence for this is growing as reviewed below.

The advent of COVID-19, and our local responses to it, have illuminated an outpouring of compassionate courage, a sense of interdependence, self-sacrifice and helpfulness. In addition, it has raised the issue again that many of us want a more compassionate, cooperative world, whilst recognizing the means to achieve this is unclear and fraught with problems, one being callousness. This paper will use an evolution informed, biopsychosocial lens to explore why moving to a more compassionate society would be beneficial for our physical and mental health, social justice, productivity, and prosocial behavior (Staub, 2003; Gilbert, 2009; Harari, 2014; Keltner et al., 2014; Kasser, 2016; Ekman and Ekman, 2017; Haslam et al., 2018; Piff et al., 2018; Ryan, 2019; Wilson, 2019; Biglan, 2020; Becker et al., 2021), but also the serious inhibitors to that movement (Gilbert and Mascaro, 2017; Wilson, 2019). These are partly in our evolution and partly in our cultures and history of agricultural societies.

## The Challenges

To pursue caring and sharing, rooted in compassion as a public good, it is helpful to understand its facilitators and its inhibitors, which are many and various. Among the most daunting inhibitors include the facts that:

1. The human brain has been rather cobbled together over a long evolutionary timeframe with various trade-offs and compromises plugged in along the way (Gilbert, 1998; Nesse, 2019; Workman et al., 2020). Much of our decision-making and “urges” are not based on rational choices, but the pursuit of life tasks evolved over millions of years with deeply ingrained, socially trained motives and emotions (Keltner et al., 2018). This means that:
2. We are a species of extremes (Gilbert, 1989/2016, 2018; Marsh, 2019) with evolved strategies and potentials for helpful and prosocial behavior (Gilbert, 2009, 2020a; Keltner et al., 2014; Mayseless, 2016; Seppälä et al., 2017) but also extremely harmful, cruel, callous, and destructive behavior to self and others (Gilbert, 1989/2016, 1998, 2009; Black, 2016; Eisler and Fry, 2019; Zimbardo, 2008; Hobfoll, 2018; Nesse, 2019). If we consider our dispositions for war, slavery, torture, domestic violence, and violence as entertainment, and the ease by which we see other humans (and animals and nature) simply *as a resource* to be exploited, then with some exceptions, the last 5,000 years or more have been anything but compassionate (Rummel, 2002; Van Vugt and Park, 2009; Glover, 2012; Black, 2016).
3. Since the advent of agriculture and expanding wealth and group size, we have created social contexts that are punitive, intimidating and often threatening with aggressive dominant males (and their small, immediate support networks) subjugating, exploiting, suppressing subordinates, and waging wars, along with colonial exploitation of other societies and cultures (Black, 2016).
4. As part of that legacy, the creation of storable resources and wealth expansion generated by agriculture created intense competition resulting in huge disparities in wealth distribution and social power which today is extreme and damaging (Wilkinson and Pickett, 2010; Stiglitz, 2012; Harari, 2014).
5. Since all dominant elites seek to protect their privilege and advantage, we also live in a world where today, the powerful and wealthy elites have disproportionate political power that has allowed them to undermine democracies and movements to social justice and fairness (Chomsky, 1992; Lipman-Blumen, 2005; Gilligan, 2011; MacLean, 2017) with continued use of violence and the threat of violence to maintain power, and exploit natural resources in extremely harmful ways (Pilger, 2007; Gilligan, 2011; MacLean, 2017; Hickle, 2018; Piff et al., 2018).
6. As people become wealthier, they become less compassionate not more (Van Kleef et al., 2008; Piff et al., 2018). After a certain point, wealth changes us psychologically and physiologically such that our feelings of compassion and empathy go down (in other words we become more callous). At the same time we shift to a sense of entitlement, deservingness, and self-interest competitiveness and promote *control and hold* (not care and share) attitudes to resources. Wealthier individuals are more likely to cheat and deceive (Piff et al., 2018). In fact, the cheating and manipulation of the wealthy (e.g., tax avoidance and back room deals, use of the media) is a serious issue for compassion. We are becoming more narcissist and self-focused, not less (Twenge and Campbell, 2009) and our orientations to compassion (at least before COVID-19) are reducing, not increasing (Trzeciak and Mazzarelli, 2019; Harvard Graduate School of Education, 2020).
7. Because our society needs to promote the production of goods and services as a source of employment and wealth creation we need to consume them and have become more materialistic. Materialism clearly supports the *control and hold strategies* to resource seeking and distribution but not without personal and social cost. Kasser (2016) points out “that people who place a relatively high priority on materialistic values/goals consume more products and incur more debt, have lower-quality interpersonal relationships, act in more ecologically destructive ways, have adverse work and educational motivation, and report lower personal and physical well-being. Experimentally activating materialistic aims causes similar outcomes” (p.489).
8. In a recent major meta-analysis of a large number of personality traits linked to happiness and well-being, Thielmann et al. (2020) found the top traits were: trust, social value orientation, guilt-proneness (being sensitive to hurting others), honesty-humility, pro environmentalism, and concern for others. The traits that were least associated with happiness were those typically linked into the competitive orientation to life. These were: narcissism, envy, social dominance orientation (SDO), competitiveness, greed, psychopathy, Machiavellianism, and sadism. Given that the dark triad traits of Machiavellianism, psychopathy, and narcissism are overrepresented in the higher-levels of politics and business, one can see the social-wide problems we need to deal with (Furtner et al., 2017; Peterson and Palmer, 2019).
9. While many espouse compassion as a moral value, individuals can also be fearful of it, resistant and block it (Gilbert et al., 2011; Gilbert and Mascaro, 2017; Kirby et al., 2019). Those with ruthless ambition, narcissistic traits and are hyper-competitive are particularly resistant to having compassion for others (Basran et al., 2019).
10. We remain uncertain how to address the disproportionate power of the few who have tendencies to drive the serious dark side of humanity (Bakan, 2012; Black, 2016; Gilbert, 2018; Wilson, 2019). At an evolutionary strategic level, our brains are the battle grounds for two very different reproductive and resource distribution strategies which are *caring and sharing versus controlling and holding* and even our phenotypes may be different according to which of those gains ascendancy (Lepage et al., 2020).

The bottom line is that our extraordinary potential to be a caring species (Keltner et al., 2014; Ricard, 2015; Narvaez, 2017, 2020; Spikins, 2015, 2017) evolved in small groups where everybody knew everybody, resources were relatively scarce, and caring and sharing those resources was essential for survival and cementing affiliative relationships (Fry and Söderberg, 2014; Lavi and Friesem, 2019; Wilson, 2019). There is now considerable evidence that our brains and bodies “work best” in environments where we feel cared for and about, and feel we can make a contribution that others value (Gilbert, 2009; Brown and Brown, 2015, 2017, Seppälä et al., 2017). Nothing prepared us to deal with the consequences of developing agriculture and creating environments of expanding groups, the need to work long hours tending fields and farms, and opportunities to acquire, accumulate and store vast resources. Overtime we drifted into a very different type of resource regulation strategy, giving up a caring sharing lifestyle and replacing it with a resource owning, accumulating and power seeking, hierarchical one (Harari, 2014). Tragically for our species, this facilitated the re-emergence of the aggressive dominant male hierarchies who were able to stimulate highly destructive competitive behaviors, increased dispositions to callousness and cruelty, and maintain power through threats and terrors.

If we are to create a more compassionate world, that serves the common good (Ekman and Ekman, 2017), then much depends on how we formulate, contextualize and think about two issues. (1) Be much more engaged with the suffering in the world and how to address it, and as part of this (2) Address the challenges of “resource caring and sharing” versus “competitive controlling and holding” (Piff et al., 2018). We will need new ways to think about how we can create social contexts that fit our needs for cooperation, caring and sharing to address many of the serious problems we face; climate breakdown, disease control, economic inequality and the need for social, gender, and racial justice (Helliwell et al., 2012; Wilson, 2019). We vote for leaders who appeal to our own desires to control and hold rather than care and share and therein lies one of our difficulties (Lipman-Blumen, 2005; Mols and Jetten, 2020). Compassion, caring and sharing will require sacrifice of some of our personal material wealth for social wealth and health. To pursue this endeavor we can explore the evolution and dynamics of caring, sharing, and compassion, how we evolved the potential to be a compassionate and courageous species but also the nastiest, vicious and cruel (Gilbert, 2005, 2018; Black, 2016).

## Origins and Flows of Caring and Compassion

Research in the field of prosocial behavior and compassion has accelerated greatly in the last 10 years (for reviews see Seppälä et al., 2017). As a result Mascaro et al. (2020) highlight the fact that there are now slightly different conceptions of compassion and different ways of measuring it. Nonetheless, all recognize compassion is about our orientation to be helpful rather than harmful, and empathically engaged rather than callously indifferent to the pain and suffering of self and others. One evolved route to our motives and competencies to be compassionate is from phylogenetically ancient forms of caring for offspring (Gilbert, 1989/2016; Geary, 2000; Gilbert, 2005, 2020a,b, Goetz et al., 2010; Brown and Brown, 2015, 2017; Mayseless, 2016; Carter et al., 2017). One function of parental caring is to be sensitive to distress and needs of offspring and try to address them. Hence, we can define one function of caring and its “derivative” *compassion* quite simply in terms of its underlying motivation and “*if* A *then* do B” algorithm where “A” would be some signal of distress, suffering or need and B would be the response and actions to address them (Gilbert, 2020a,b). This gives rise to defining care and compassion as “*a sensitivity to suffering in self and others with a commitment to try to alleviate and prevent it*” (Gilbert, 2017b,c). Needs are important in compassion because if they are not addressed suffering will clearly follow (Gilbert and Choden, 2013).

Like compassion, *callousness* can have slightly different definitions and is used in slightly different ways (e.g., in forensic settings) but here I define it as the opposite of compassion; as an *insensitivity to suffering and an indifference to its alleviation or prevention* (Gilbert, 2005, 2018). In addition, we can see these as dimensions and contextually influenced. We can be compassionate in some contexts but callous in others and the degree and intensity of compassion and callousness can vary too. Poulin (2017) also reviews evidence where we can be very sensitive to suffering but actually not do anything about it. How that falls on the compassion callous dimension is unclear.

As a motive, compassion is not dependent on emotion. Whether you are an *anxious* firefighter risking your life to save others, an *angr*y fighter of injustice or experiencing *sadness* as you counsel the bereaved, all these have one motive in common: the motive to turn toward suffering or need, be prepared to develop *the courage* to experience the threat, distress or pain involved, and develop *the wisdom* of working out what would be helpful. Sometimes compassion can be a logical or moral choice as much as an emotional one (Loewenstein and Small, 2007). And our reasons for *behaving* compassionately may have multiple sources both conscious and unconscious (Böckler et al., 2016; Bargh, 2017). Despite these complexities core processes of compassion show up in physiological and psychological domains.

### Physiologies of Caring

Over millions of years, in many species, care given and received evolved to have major impacts on the functioning of the autonomic nervous system, the immune and cardiovascular system, and neurophysiological (brain) circuits that play fundamental roles in self-identity and self-experience, emotion regulation, and prosocial or anti-social behavior (Gilbert, 1989/2016, 2017c; Keltner et al., 2014; Music, 2014, 2017; Brown and Brown, 2015, 2017; Mayseless, 2016; Carter et al., 2017; Porges, 2017; Seppälä et al., 2017; Stevens and Woodruff, 2018; Di Bello et al., 2020; Kim et al., 2020a,b). Relationships are physiological regulators (Hofer, 1984, 1994). Changes to the autonomic nervous system, particularly the myelination of the 10th cranial nerve of the parasympathetic system to become the vagus nerve, played a major role in how the caring relationship came to regulate threat processing (Porges and Furman, 2011; Porges, 2017). It now looks as if the parasympathetic *rest and digest system*, which regulates (sympathetic) threat and drive states, was incorporated into the systems for close-relating thereby enabling the signals emanating from a parent (caring other, close friend) to have soothing, vagal-mediated qualities on an infant (Porges, 2017). One measure of the efficiency of the vagus nerve is called heart rate variability. There is good evidence that the functioning of parasympathetic system as measured by heart rate variability plays a major role in prosocial behavior and caring and compassion in general (Keltner et al., 2014; Petrocchi and Cheli, 2019). In contrast poorer functioning of the vagus nerve and balancing of the autonomic nervous system, as measured with lower heart rate variability, is associated with less cortical control over basic emotions increased vigilance to threat, aggressiveness and decreased prosocial behavior (Lepage et al., 2020). As noted below these distinctions are important when we come to explore variations between sharing and caring strategies and controlling and holding ones.

Porges (2007, 2017) has written extensively of how the vagus nerve became part of a circuit that was very sensitive to facial expressions and voice tones, particularly those indicating friendliness and safeness. Indeed, today, the way we look at, share facial expressions and play with children all indicate our intuitive understanding of how our voices and facial expressions impact on them. We change these signals when we are engaged with adults, but nonetheless, we intuitively understand that friendship signals (and lack of it) are conveyed through voice tones and facial expressions.

The hormones oxytocin and vasopressin also played a crucial role in the evolution of caring behavior for infants, pair bonding and close friendships (Carter et al., 2017). In fact, there is now considerable evidence that kindness and compassion from others, when we are under stress or experiencing losses have significant emotional and physiological regulating effects (Steinbeis et al., 2015; Cassidy and Shaver, 2016; Mayseless, 2016; Seppälä et al., 2017). Rockliff et al. (2011) found the nasal oxytocin made it easier for people to imagine a caring other being caring of them. Morhenn et al. (2012) found that receiving touching massage reduced stress hormones and increased oxytocin. In social mentality theory (Gilbert, 2017a), both the giving and receiving of signals impact similar systems. Hence we are biologically set up with systems designed for the giving and receiving of care.

In addition, priming people with memories of caring others or seeing caring behavior can impact on physiological systems involved with stress and the way people cope with stressful events (Norman et al., 2015). Individuals who sense themselves as living in supportive, caring communities are in different physiological states than individuals who are in threat-focused communities or see the world as “a dog eat dog” place where you cannot trust or rely on others to help you (Perry et al., 2013). The general sense of being contextualized in caring and socially safe versus socially unsafe environments may well be crucial for many psychological processes (Kelly et al., 2012; Armstrong et al., 2020). Variations in the oxytocin gene may also link to variations in compassion and prosocial behavior versus callousness (e.g., Tost et al., 2010; Marsh, 2019). If watching others being caring or being harmful has such powerful effects on us, then we really do need to take another look at our entertainments that more commonly depict characters as narcissistic, argumentative and aggressive rather than fun, loving and friendly.

### The Phenotypic and Epigenetic Effects of Caring

Epigenetic science is important because it helps us understand how different contexts result in different trait phenotypes. For example, all humans are motivated to form some kind of attachment with their caregiving figures early in life. However, the way they experience them e.g., as caring, neglectful, or abusive has profound effects on the attachment phenotype(s). Some will grow to be trusting and secure in their attachments, others are anxious and vigilant to rejection; yet others become dismissive and avoidant of close interdependent relationships. In other words, social experiences shape phenotypes via epigenetic effects which in turn impacts the maturation of numerous physiological systems (Cowan et al., 2016; Slavich, 2020). So important is received caring behavior that it actually impacts epigenetic profiles even in fish. McGhee and Bell (2014) studied three-spined sticklebacks where fathers provide the care and protection. They note that:

During the approximately two weeks that fathers provide care, they defend their nest from predators, fan the nest with their pectoral fins to provide fresh oxygen to the embryos and once the embryos hatch, retrieve fry that stray from the nest. During this period, offspring rely on yolk reserves provisioned by their mother prior to fertilization. Fathers do not feed offspring, but there is evidence that offspring antipredator behavior …., mate preference …. and morphology …. can be sensitive to the effects of fathers. (p. 2)

They go onto discuss how paternal caring influences traits, such as anxiety in offspring, that impact on their survival and how paternal caring influences the epigenetics of their offspring. Indeed, it is now known that across many different species the quality of parental caring impacts epigenetics and can attenuate or amplify vulnerabilities to threat sensitivity and sociality (Cowan et al., 2016; Music, 2017; O’Donnell and Meaney, 2020; Slavich, 2020). But it is not just the care received in the parent infant dyad. There is also increasing evidence that social contexts, particularly contexts of poverty and stress, can have long-terms epigenetic effects (McDade et al., 2019). Rightly or wrongly those favoring intense lockdowns have paid little attention to the epigenetic effects on children growing up in stressed families, social disengagement, and lacking education. Concerning too is increasing evidence that epigenetic changes can be inherited and passed to subsequent generations (Cowan et al., 2016; O’Donnell and Meaney, 2020). The implications of this are profound and still to be fully acknowledged because it means that the societies that we have been creating since agriculture may well have been affecting our epigenetic profiles. We are faced with the possibility that different cultures create different phenotypic patterns in the individuals living within them. Hence, for example, it is possible that Roman societies that accepted gladiatorial games and the harsh use of slaves were indeed phenotypically different to us.

### A Challenge: The Expense

There is however, a rather a big hitch to the evolution of caring and sharing. They work well in reciprocating groups of familiar others but did not evolve as a general giveaway. The problem with all motives, including caring motives, is that they are “energy” expensive and costly. Evolution has therefore built in its own criteria for dispensing it, sometimes referred to as different forms of altruism (Burnstein et al., 1994) and reciprocal altruism (Gintis et al., 2003; Colqhoun et al., 2020). Mothers of most species will only care for their own infants, not for others. In the case of sheep, for example, it can be very difficult to get a mother to adopt an orphan. While adoption does occur in mammalian species, it is rare; humans, who foster and carry out other forms of caring, are exceptional in this regard (Spikins, 2015). The evolution of the genetics of caring predisposes us to focus on those closest to us. For example, imagine we go to maternity to have our baby delivered and they take our baby away to weigh it and come back and say, “we have some good news and some bad news.” The bad news is we have lost the name tags and are not sure whose baby belongs to which parents. But the good news is the babies born this morning are in perfect health, so please feel free to choose the one you fancy.” Clearly, this would be a source of immense distress because we are programmed to care for our own genes, not strangers. Furthermore, if we have an opportunity to save our own children or 40 children down the road, who are we going to save? Also if we have opportunities to lavish vast resources on our own children at Christmas or send that money to children who are starving or lack clean water, who are we going to choose? It is not impossible for us to overrule these evolutionary preferences, but we need to understand them and what we need to do if we want to overcome them (Geary, 2000).

Although oxytocin is often regarded as a hormone important for bonding, it is also a hormone that makes mothers aggressive to potential threats to their offspring (Carter et al., 2017) and can power outgroup (defensive) aggression too (De Dreu et al., 2011). Evolution has built-in tendencies for us to be interested in caring and sharing with those that we are likely to know and have a reciprocal relationship with, those we like or trust, rather than those we do not. In addition, oxytocin can make us more sensitive to suffering for in-group members, but this has a potentially tragic downside. It can also make us more vengeful to those who have harmed our group (Han et al., 2020), and group vengeance is responsible for considerable violence. Physiologies do not point to simple solutions.

If we are going to create a more sharing, caring and compassionate world, we need to understand these inhibitors blocking our orientation to compassion (Gilbert and Mascaro, 2017). This is where we need to use our intelligence and override emotional dispositions or prejudices. Loewenstein and Small (2007) argue that if we rely solely on our emotions of sympathy and ability to empathize, we will exercise only limited compassion. Compassion has to be powered in part by moral values and understanding its value, not just our innate reactions. It also has to be socially valued and socially reinforced through cultural norms and expectations. Just as we can have a sense of biological kinship, so we can have a sense of psychological kinship. We are more likely to be caring to those who we see in that category. Importantly, as some spiritual traditions encourage, we can train our brains to conceptualize humanity as part of our “kin group” (Bailey, 2002). Right wing politicians however are more interested in splitting and setting one group up against another.

Another aspect of compassion is that it is linked to altruism. Altruism requires individuals to be prepared *to make sacrifices* to help people. Helping people that does not have a cost or can benefit you in the long term is questionable as to how altruistic it is (Colqhoun et al., 2020). Interestingly, therefore although helpful behavior has been observed repeatedly in young children Green et al. (2018) investigated how helpful they would be if there were a cost. They found that children would help a hand puppet achieve a goal of completing a task (e.g., puzzle) if there was no cost to them, but helping fell away significantly when they had to give up something to help the puppet. Even when the puppet made appeals and was clearly distressed, the child still would not give up their own resources or rewards to help the distressed puppet.

## The Psychological Functions of Caring

Having looked at the physiological and epigenetic effects linked to evolved caring motives, we can now turn to the *psychological functions of caring*. Parents provide many resources to their infants including food, thermoregulation and protection. In addition, they offer major psychological supports. One of the major models for understanding these supports has been attachment theory (Bowlby, 1969, 1973, 1980, Cassidy and Shaver, 2016; Music, 2017). This describes how parents provide their offspring with a *secure base* from which the young can begin to play, explore, and return to for guidance and support. Second, they provide a *safe haven* acting as soothing and comforting stimuli when the young are distressed or needy, thus helping to regulate the infant’s need/frustrations, arousal and emotions. Third, the child and parent seek to maintain proximity to each other and when separated become concerned and seek each other out (Cassidy and Shaver, 2016). As noted, these experiences can also profoundly influence the maturation of physiologies and epigenetic profiles.

Every time the child experiences others as caring, encouraging or soothing to them, they learn to turn to others when in distress. The regular experience of these means that children develop trust in others and an interest in relating to others. In addition, when caring parents show pleasure and joyfulness in their child, the child experiences themselves existing positively in the mind of others and becomes attuned to the environments their parents provide. These processes carry powerful epigenetic and physiological effects as noted above (Cozolino, 2014; Music, 2014, 2017). To put this another way, evolution has created organisms, such as ourselves, who fine tune their epigenetics and physiologies (phenotypes) according to the niche they are going to have to operate within. Put crudely, developing affiliative, trust, and dispositions to be sharing will not be helpful if the environment is going to be competitive, callous, full of down rank threats, and cheats.

**FIGURE 1:**
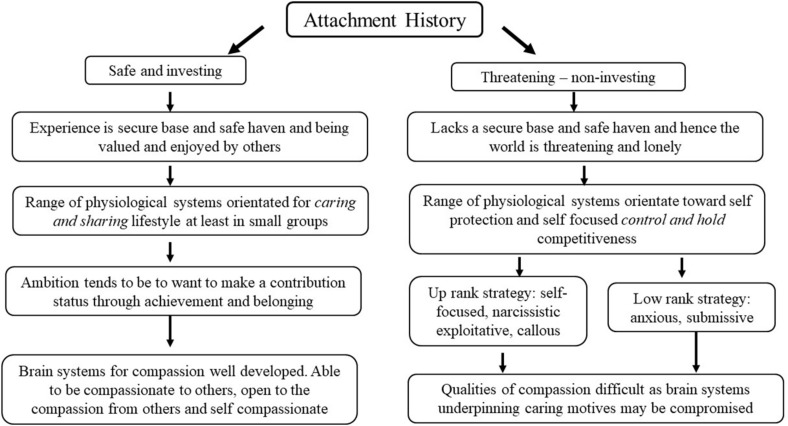
Early life background and strategic orientation: Adapted from Gilbert (2005).

Tragically when these important *brain nutrients* of care, compassion, and love are not received, and children are on the receiving end of low warmth, neglect, or abuse, it has devastating impacts on the brain, epigenetics, and subsequent development (Lippard and Nemeroff, 2020). The suffering of the Romanian children in orphanages tragically brought home just how important affectionate caring was to the development of the brain (Chugani et al., 2001). In fact, there is now considerable evidence that our basic strategic orientation (particularly on the dimensions of “helping and concern for others” *versus* “lack of concern for others”) is partly set in our life histories (Del Giudice, 2016). This is set out in Figure 1.

Figure 1 is referring to dimensions, not absolutes which can be fluid and change over a person’s life or from situation to situation. On the left-hand side, cared for children experience their brains and minds developing and orientating to a range of motives, emotion, dispositions, and competencies to pursue a caring and sharing lifestyle. On the right hand are children growing up under threat (lacking a secure base and safe haven). They find their minds developing strategies for a very different type of life and social niche; for a more “hard fought” life; a competitive lifestyle where one is wary of people and certainly cannot trust others to be helpful, sharing or caring. In this social niche, there are two strategic options (Del Giudice, 2016). Many primates (although much less so bonobos) living in hierarchical groups show that under competitive pressure there is a branching into two different strategies. One is to adopt a ‘damage limitation, lower risk of injurious conflict, better safe than sorry, a down rank type strategy of submissiveness, and lack of confidence.’ This increases the risk of depression and anxiety. It may fluctuate somewhat depending on how secure and safe subsequent relationships are, but these individuals can be very sensitive to feeling failures and inferior compared to others, socially marginalized and often carry a sense of loneliness and disconnection (Gilbert, 2000, 2020a). These individuals can be caring, but this can be because they want to be liked and avoid rejection (Catarino et al., 2014; Böckler et al., 2016).

The other strategy in contexts of low care is a riskier up rank strategy, to really push one’s luck, display confidence (perhaps even grandiose), seek power and accumulate resources, foregoing sharing caring strategies. These are linked to certain types of narcissism and psychopathy both of which involve toning down caring and sharing motives (Del Giudice, 2016). One version links to a sense of entitlement to treat others as a resource, and in the extreme regulate their relationships through fear. Many of the people in our prisons did not have good starts and their capacities for empathic connectedness to others is limited. It is also the same for some of our politicians and the higher up you go in business, the more you will find the rank focused callous folk who can be superficially charming, but have little interest in caring and sharing (Furtner et al., 2017; Peterson and Palmer, 2019). Can these individuals be turned to change these fundamental evolved strategies that are running their show? It is unclear, but there are glimmers that compassion training, which involves specific techniques to stimulate care-focused social mentalities, by helping people process some of the neglect and harshness they themselves experienced, is proving an exciting potential intervention for forensic youth (e.g., Ribeiro da Silva et al., 2019, 2020). Shirtcliff et al. (2009) show that individuals who are callous are not emotionally in tune or physiologically responsive with their own emotional pain. Hence, physiological systems involved with “sensitivity to emotional suffering” are “offline,” meaning that because they are not in tune with or responsive to their own emotional suffering they unable to be sensitive to any signals of pain including those they cause others. However, keep in mind that mentalities are co-regulating and therefore if individuals can start to process their own pain and their unmet needs to have been cared for, this *might* open up that social mentality. It does not open up unless those *inner care receiving systems* are worked with; trying to help them feel a sense of remorse or guilt when their own emotional pain systems are closed down may fail. Indeed Compassion Focused Therapy (Gilbert, 2010, 2020a), is less focused on changing basic beliefs and much more interested in motivational and phenotypic switching, along with building physiological pathways and networks that facilitate the caring social mentalities (Gilbert, 2017a, 2020a,b). The degree to which that is possible is the next major challenge in psychotherapy development and research (Cozolino, 2017; Gilbert, 2019b,c, 2020a).

There is however a twist to this and that is that being born into wealthy parents is also associated with the forms of narcissism that can make individuals unsuited as leaders (Martin et al., 2016). It is unclear if this is a direct effect of wealth or the fact that wealthy parents have different types of relationships with their children. There is some evidence to suggest that wealthy parents find parenting less meaningful and maybe less emotionally engaged with it preferring money making (Kushlev et al., 2012). Wealthy parents are more likely to send their children to boarding schools with negative consequences (Schaverien, 2015). Parental emotional distancing maybe the issue. Then there is the well-known narcissism that comes from simply being spoilt and having few boundaries put on them; growing up completely disengaged from the experiences and the realities of poorer people for example. Living in a world of plenty may not support developing empathy for suffering because one rarely encounters it; or if one does, it tends to be trivial, like having a tantrum because one cannot have the size of birthday party one wanted. Wealth may be a hindrance to developing empathy for suffering (Piff et al., 2018).

Whatever the origins of narcissism, the human brain *needs considerable stimulation of feeling safe and cared for along with developing respect and concern for others to mature into the kind of ethical caring and sharing individual we so need as a species* (Gilbert, 1989/2016; Slavich, 2020). Nothing is inevitable and of course people from difficult backgrounds can become wonderful examples of compassion and people from good backgrounds can become villains. Nonetheless, the extraordinary way in which our bodies, genetic profiles and minds are so linked to our environments is a message that politicians are still ignoring probably because the cost of really taking this on board is evaluated by them to be high. Tax cuts rather than children’s brains are more important. Prosocial and antisocial tendencies are potentials within all humans to some degree or other, give or take a little genetic variation here and there. *Individuals simply find themselves maturing into these strategies in response to the relationships in which they are embedded.* Children do not wake up one Saturday morning and choose which one they are going live by; that they could be an altruist but then think “that is boring I will train to be a narcissist.” It is all about how our brains and epigenetic profiles *automatically pattern* themselves in different environments (Del Giudice, 2016).

### From Caring to Compassion

Species with high levels of parental caring *evolve complex cognitive competencies*, whereas those that do not, do not (Uomini et al., 2020). Over time, these cognitive competencies change the way basic motivations are experienced and expressed. Certainly, by the time of homo sapiens, the potential to use our reasoning minds for inhibiting or accentuating a motive is extraordinary (Byrne, 1995; DeFelipe, 2011). We become capable of orientating caring toward medicine, developing anesthetics, antibiotics, ridding the world of smallpox, and extraordinary keyhole surgery. Unfortunately, the same competencies can be used for planning wars, building nuclear weapons and designing new tortures.

We have at least three different types of cognitive competencies that give rise to different insights and wisdoms (Gilbert, 2019a, 2020a).

1. **Reasoning insights:** First, we have certain *types of reasoning* that enable us to understand and have insights into complex causalities, system relations and ‘how things work.’ We can imagine and run what if… and suppose/imagine that… simulations in our mind. We can think about the past and future and plan, not just days, but years ahead (Suddendorf, 2018; Baron-Cohen, 2020). And we accumulate knowledge at an increasingly exponential speed so that we can anticipate that our science will be very different in 100 years to what it is now. This is the basis of the scientific mind that put people on the moon.
2. **Empathic insights:** Empathy is a competency, not a motive (Gilbert, 1989/2016; Decety and Ickes, 2009; Baron-Cohen, 2011). It can be used by any motive. If you want to manipulate somebody, if you want to be good at developing new relationships or dating, you are much more likely to be successful if you are empathic, than if you are not. While empathy certainly helps caring behaviors we may not need to understand the exact nature of somebody’s suffering to want to help them. Whether men and women can understand what it is like to be the other gender (e.g., have a baby) is uncertain; the degree to which white middle class people understand experiences of racism is also uncertain; the degree to which white middle and upper class politicians understand the real daily struggles, fears and despairs of the poor is uncertain. Nonetheless, in these situations we can be empathic to the fact that we might struggle with empathy and therefore we need to listen and learn and not make assumptions about our understanding. It is the motivation to be caring that is crucial. Without a caring motive empathy can be used for selfish, deceptive, and manipulative goals (Bloom, 2017).
3. **Consciousness of consciousness insights:** Third, we have a new type of *consciousness of consciousness:* we can be aware and know that we are aware. This facilitates extensive self-awareness and opportunities *to become mindful and observant of our own minds* (Gilbert and Choden, 2013; Brown et al., 2015). Learning to pay attention to what is going on in our minds, so that we can gain more control over its outputs is one of the great challenges of humanity. Unfortunately, without care, self-awareness can also be a curse and the source of egoism, shame, depression and narcissism and considerable harmfulness to ourselves and others (Leary, 2007).

People can be good at some of these competencies, but not others. For example, some individuals are brilliant scientists, but are very poor at empathy. Indeed, talented scientists or business people can also be somewhat lacking in empathy or be on what is called the Asperger’s spectrum, which has implications for how they understand social needs (Baron-Cohen, 2011, 2020). Some politicians may have these tendencies too. Others can have excellent empathy skills, but they will never be able to win a Nobel Prize for physics, be entrepreneurs or make good politicians. Some individuals can be very bright, but not have much insight into their own minds or an ability to be mindful; whereas mindful people are not necessarily the most empathic, intelligent or caring. Indeed, recent research has shown that mindfulness, empathy and compassion skills can be trained differently and influence different brain systems (Singer and Engert, 2019). Training our minds for global compassion, therefore, is going to be a multi-focused task (Dalai Lama, 1995; Ekman and Ekman, 2017).

**FIGURE 2:**
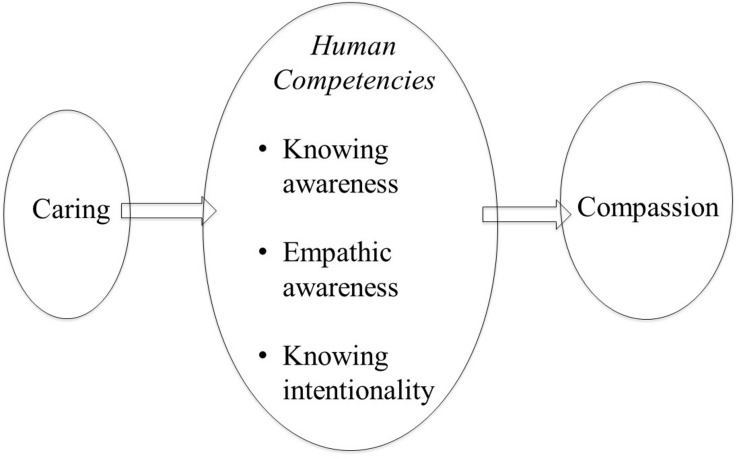
From caring to compassion: from Gilbert (2018). Living like crazy with permission Annwyn house.

Collectively these competencies give rise to what we can call *knowing intentionality* (Gilbert, 2018, 2020a). It is doubtful whether any other species can engage motives knowingly in this way. Lions clearly intend to hunt and kill their prey, but not with self-aware *knowing* awareness. They cannot suddenly decide *not* to do it, become vegetarians or to lose weight. *Knowing intentionality* has probably been one of the most important human competencies that have powered science and built the cultures we have. It is *knowing intentionality* and the use of these human cognitive competencies that turns basic caring motives into compassion. This is depicted in Figure 2.

The domain of *knowing intentionality*, however, is also tricky because many of the reasons we behave in the ways that we do are partly unconscious (Huang and Bargh, 2014; Bargh, 2017). Nonetheless, humans do have opportunities to gain insight into the nature of “mind” (Baron-Cohen, 2011; Siegel, 2016) and then choose to develop compassion motives and a compassionate identity; to live to be helpful, not harmful (Dalai Lama, 1995; Gilbert, 2009; Ricard, 2015).

A further distinction should be made. Caring can be used for many non-sentient objects and beings, whereas compassion cannot. For example, we can talk about caring for our gardens, homes, cars or prized possessions, or our planet, but we do not use the term compassion for that type of caring because these are not sentient and do not experience suffering. Hence, the root of compassion is in the caring motives, textured by our unique minds (DeFelipe, 2011) and *awareness of suffering* (Gilbert, 2009). So, this brings us back to the fact that compassion is the *intentional* desire to be “sensitive to suffering in self and others with a commitment to try to alleviate and prevent it” (Gilbert, 2017a). Many animals may care for their young or others, and ants may carry their injured colleagues back to the nest (Kessler, 2020), but only humans as far as we know have this *knowing intentional awareness* and can develop deep wisdom of how to be helpful – can develop science and medicine or seek the politics of caring. This is profoundly important because it means that compassion is an intentional desire to bring caring into our relationships, into our world, into our politics, into our businesses, and we can use science and research of how best to do those. Using our new intelligence can change our social discourses and thereby over time change our phenotypes.

### The Flows of Compassion

Importantly too compassion has a three-way orientation. In addition to giving care to others, being compassionate with oneself and *being the recipient of compassionate care*, resource sharing and support has many profound impacts on us psychologically, physiologically and our social behavior. The quality of caring we receive, from the day we are born to the day that we die, has a huge impact on many indicators of physical and mental health, longevity, and prosocial behavior (for major reviews see Thayer et al., 2012; Seppälä et al., 2017). Being the recipient of, and open to, emotional and compassionate support from others has long been linked to a buffer against mental health problems (Hermanto et al., 2016). Remembering or bringing to mind individuals who are caring and supportive helps people cope with stress (for review see Norman et al., 2015; Gilbert, 2020a). Although there are different definitions of self-compassion there is now substantial evidence that self-compassion and self-reassurance buffer against depression and facilitate coping with setbacks and life difficulties and promote wellbeing. In contrast, harsh self-criticism, fears of being self-compassionate and fear of accepting compassion are associated with increased mental and physical health problems (Kirby et al., 2019).

Last but not least, as emphasized repeatedly in Buddhist teachings (Ricard, 2015), and confirmed by considerable research, the more we orientate ourselves to be helpful to others rather than self-focused, the happier and healthier we are (see Crocker et al., 2017; Seppälä et al., 2017). In addition, general fears and resistances to being compassionate to others is associated with hyper competitiveness, narcissism and ruthless self-ambition (Basran et al., 2019); traits not untypical of power seekers (Peterson and Palmer, 2019). In a major review on what they call motivated “otherness” or “selfishness” Crocker et al. (2017) highlight how being compassionate to others, and feeling one has a contribution to make, has major emotional and physical benefits in comparison to self-focused competitiveness. Hence, the evidence for all three flows of compassion is that they are profoundly important for our physical, mental and social well-being.

Despite this, many individuals perceive compassion as a weakness or softness and rather than promote compassionate values for society, promote competitive self-interest (Sachs, 2012). We are at a point now where it is not a matter of political opinion or preference but creating our societies for the common good should be based upon the science of what creates it. One of the problems with understanding compassion and how we miss its fundamental attributes of complex variations according to context, and the central role of *courage and wisdom* is that it is often confused with other prosocial behaviors. For example, compassion is not the same as kindness because compassion involves degrees of courage and wisdom in ways that kindness does not; the emotions associated with kindness tend to be positive whereas those associated with compassion are more difficult; and compassion often involves sacrifice (Ricard, 2015). For example, this is not a religious point but we talk about the compassion of Christ not the kindness of Christ (Gilbert et al., 2019). Nor is compassion the same as the western concept of love with which it is sometimes confused. The strongest forms of compassion are for people we do not love or even like. And compassion is sometimes confused with submissiveness that we simply have to accept or give in to harmful behavior. Nothing could be further from the truth. Indeed, in a well-functioning society one would have a well-functioning “compassionate” police force and an agreed set of laws. Without agreement on what we mean by compassion it is going to be tricky to have it widely adopted particularly for those individuals who think it is somehow a costly nicety, soft or weak when in reality it is the most morally wise and courageous of all of our motives (Lampert, 2005).

### Summary

With these insights and concepts on compassion we can see how compassion *is profoundly important to so many aspects of our lives;* texturing epigenetic profiles, multiple physiological systems and psychological values plus how we relate to each other. Consider the passions of people engaged in charity work all over the world, building wells for freshwater, making medicines available to the poor, or in international peace efforts trying to end the conflict and release perhaps millions of people from the intense suffering of the conflict. Consider a recent movement of “compassion in politics” which seeks to stop politicians passing laws that are known to cause suffering to the least able to cope (see below). Note too that some people can be very impassioned for some domains of caring and compassion but not all; motives can be specific. And some people can be very good in some aspects of compassion but not others. Our courageous firefighter might not make the most empathic partner or parent and an empathic counselor may not make the best firefighter or international negotiator. Many different talents therefore can be utilized to work in different contexts and with different types of suffering.

## A Multi-Mind of Conflict and Uncertainties

### Motives, Emotions, and Algorithms

One theme of this paper is that to understand and promote compassion requires us to step back in time, look into how the brain evolved and gain insight into its multiple motives and algorithms that can conflict and be difficult to regulate in the modern world. We can start by noting that organisms evolve because they confront and adapt to the two challenges of life of *survival and reproduction* (Buss, 2019; Workman et al., 2020). These challenges give rise to all living things that face *three basic life tasks*. They have to be able to identify and *defend themselves against threats* in their domain of existence. They have to be able to seek out the *resources that will sustain them* and help them to reproduce. Third, they have to be able to identify when they are *not* under threat and have sufficient resources to allow them to *rest and digest*. Ways of *building a compassionate society will have to address how we go about all three basic life tasks and motives* because they are the ones that are involved in both control and hold and care and share. To state it briefly and somewhat simply, control and hold have both elevated threat linked to losing control over resources and elevated drive to obtain them putting their own security in their own hands. Care and share are seeking a more hunter gatherer lifestyle where the joys of life and security are in the hands of others. Especially important will be helping people experience emotional states of contentment and connectedness rather than feeing a need for ‘more and more’ (Naish, 2008). We can look at each of these in turn.

Threat, protection/defense motives are run by threat based algorithms of, *if* a threat is detected *then* (for example) stimulate the sympathetic nervous system, increase heart rate, focus attention and then run like hell (or perhaps engage in a fight). We now know quite a lot about the physiology of the threat algorithms and how they can be primed, textured and regulated through early life experience and social context. In addition, there are specific emotions that support this motive. These are: anger (fight), fear/anxiety (flight), and disgust (expel). When threats are overwhelming and cannot be controlled animals and humans can go into shutdown depressed states (Gilbert, 1992/2016, 2020c). Our thoughts, beliefs and values can also regulate our sensitivity to threats and the actions we take. We can greatly amplify, ruminate and stimulate our threat systems by what we think but we can also attenuate and override them. We might feel very anxious taking a driving test, but we overrule that anxiety and take it because we have a high motive/desire to drive. Indeed, the regulation of emotion, particularly potentially harmful emotions, is crucial to maturation and wellbeing. So we might feel desires to be selfish or even harmful but we can override them with sufficient insight and compassionate intention (Loewenstein and Small, 2007; Ricard, 2015).

One of our most important insights is that the threats we are faced with today are very different from the hunter-gatherer threats. Those threats, of having sufficient to eat for example, were solved with caring and sharing. Today however, we are unable to hunt and gather and are totally dependent on work, the making of objects and things that may have very little personal relevance only to provide money to gain access to resources. And we have to work to please our bosses or to meet the requirements of the job (deadlines and work flows) which puts us under constant vigilance of judgment by others who may deem us worthy of promotion or demotion. Agriculture increased the amount of time spent in work (Harari, 2014) and so today we have far less time for family and socializing than working (Bunting, 2004). So today our lives are considerably more chronically stressful and threat focused but in very different ways to a hunter gatherer lifestyle. For many centuries the threat and stress of trying to survive in these artificial environments has been intense and we now know that these types of stresses had major impacts on physiological systems. In addition they make us less charitable, less compassionate and more self-focused with group favoritism and aggressive defenses (Steinbeis et al., 2015).

Indeed, there is increasing evidence that those on the right wing of the political spectrum see the world through the lens of the threat system. Right wing authoritarianism (RWA) is associated with highly threat sensitive perception of the world even a slightly paranoid view (Wodak, 2015). We have not the space to review this literature here but can refer to one study that captures important themes of this threat. Shaffer and Duckitt (2013) found that an exploratory factors analysis identified five distinct fear–threat factors linked to RWA. These were:

Harm to Self, Child, or Country; Personal and Relationship Failures; Environmental and Economic Fears; Political and Personal Uncertainties; and Threats to Ingroup. All the fear–threat factors were correlated with RWA, with the strongest correlations being for Threats to Ingroup, and with stronger effects for social than for personal fears (p. 6).

Although the data on early attachment experiences and these fear based orientations to the world is a little thin, it is probable that some of them were set up for this fear-focused phenotypic orientation because of problematic backgrounds. One of the most salient dimensions underpinning shifts into control and hold (and away from share and care, in both the poor and the wealthy but for different reasons) is the perception of threat. In the wealthy it is the threat of losing what they have, and in the poor is the threat of not having enough. Both types of threat sensitivity can make us manipulable to threat messages and tribal aggression (Hobfoll, 2018). Many authors have also highlighted how both politicians and the media seek to stimulate these threats as the sea for them to sail into power and create *complex interactions* between the minds of leaders and the minds of those supporting them (Mols and Jetten, 2020).

Competitive resource seeking motives focus on obtaining and gaining resources conducive to flourishing, survival and reproduction. The orientation is approach rather than avoidance and the associated emotions tend to be positive with the desire to repeat the actions. Fredrickson (2013) suggests the evolved function of positive emotions is to *broaden and build* one’s resource base. Given that, then positive drive emotions will work quite differently in hunter-gatherer societies compared to western, high resource, competitive societies. Most animals and early humans do not store or accumulate resources. Hence drive emotions were very much to secure resources for immediate use and therefore “turn off” when what is needed is obtained. The level of need was the regulator of drive and once basic needs had been met one could then focus on other *rest and digest activities*, play, creativity and socializing (Lavi and Friesem, 2019). Today we are still very motivated to socialize and to share positive (smiles and jokes) emotion, be at parties, celebrations, and to come together for joint tasks (e.g., as observers of sport, music, cinema, and so forth; hence the tragedies of lockdown). However, we are also very preoccupied with our access to wealth and resources.

The general consensus is that unlike us today, stressed out by work and the need to maintain ourselves in work, hunter-gatherers worked less, spent more time socializing, were relatively peaceful and affiliative in their relationships and more likely to experience contentment (Fry and Söderberg, 2014; Harari, 2014; Ryan, 2019; Hunt, 2020). These living styles have their own physiological profiles and supports health (Cordaro et al., 2016). In these states, the body is able to repair itself, and long term is associated with health (Thayer et al., 2009) and prosocial “care and share” behavior (Keltner et al., 2014). Agriculture however was to change all this because it introduced storage and that meant that there was no limit to drive behaviors because there is no limit to how much one could store and gain control over; “working to acquire” took on a whole new time consuming process (Bunting, 2004). And it was not long before more dominant individuals enticed more subordinate individuals to work for them. We were on an exponential curve to resource accumulation and the beginnings of the hierarchies of wealth and power. Overtime, the wealthy turned other human beings into slaves and continually drove down ‘wages’ to ensure dependency, but also excessive hours of toil to survive. Nothing like this existed before agriculture. Last but not least some of this excessive pursuit of resources is threat based, the fear of losing or falling behind (Basran et al., 2019; Jetten et al., 2020).

### Social Mentalities, Caring and the Conflicts of the Mind

It is clear that caring behavior is only one basic social motive amongst many others. Social motives have been called social mentalities because they are different to non-social motives, and can only be satisfied within an interaction; that is you need a partner to co-create them with you (Gilbert, 1989/2016, 2017a). This means they have to co-evolve. Now, of course, much in evolution is actually a process of co-evolution, such as predator-prey, disease-host. However, for social behavior, co-evolution occurs because there are benefits to that interaction *for both parties*. Sexual behavior can only evolve if individuals can both send signals and be responders to signals sent to them. There is not much point of male Robins evolving a red breast if the females are more interested in shades of purple. Sending a signal and receiving a signal in a specific mentality, in this case sexuality, stimulates the reciprocal physiological systems in participants whether the individual was first a receiver or sender. This is not to deny that nature also has a lot of coercive sex. Dominant subordinate behaviors can only evolve if dominant individuals threaten in a particular kind of way and subordinates have evolved the inner algorithms for submissive behavior, which when expressed turns off the attack in the dominant. Indeed, it is generally accepted now that it is the subordinates that regulate the hierarchies through their preparedness to submit under threat (Gilbert, 2000). Importantly, animals do not learn *how to submit*, that is part of their algorithm, but as they grow, they test the waters and increasingly, as they get stronger and bigger, fight back and challenge for resources.

An evolved social mentality that can block caring, and pattern the mind in a completely different way in terms of what it pays attention to, what it aspires to, what activates positive emotions and turns off empathy and concern for others, *is self-focused competitiveness*. This is particularly the case because part of the competitive algorithm is to suppress and inhibit the competitive behavior and claims (resource seeking, and needs) of others. Competitive behaviors vary according to whether contestants are in-group or out-group. When many primate groups meet, they can engage in war like behavior and injure each other (Bonobos are different, Tan et al., 2017). In intergroup violence submissive behavior is not that effective. We see this in humans as well. We can be vicious to out-group members regardless of whether they are showing signs of submission or not (Staub, 2003; Zimbardo, 2008). In centuries past, the vanquished were executed sometimes brutally or sold into slavery; they become an enemy to be removed or a resource to be used. As noted below, competitive behavior has that disposition to turn any other lifeforms including humans into resources to be used and exploited.

Along with care and share another counter to unregulated competitiveness is *cooperative behavior*, something humans excel at Wilson (2019), but there are different dimensions to it (Gilbert, 1989/2016). For example, the way we cooperate in our intimate relationships utilizes different physiological systems than when cooperating with strangers to produce a particular outcome or to regulate a conflict. In humans, cooperation also emerges out of an empathic understanding of intentionalities linked to intersubjectivity and trust. I climb the tree to bend the branch so you can get the apples but trust you not to “cheat” and run off with all of them! The issue of *cheating* where individuals take far more than their fair share of resources in the pursuit of *control and hold* would have been a major problem in hunter-gatherers and was very definitely shunned. However, it is quite the opposite in capitalist societies where not only is there no criteria for deciding “fairness” because that has been abandoned as a human endeavor in capitalist societies, but wealth differentials are actually status symbols and aspired to Wilkinson and Pickett (2010); Wilson (2019). Indeed, the legitimization and justification for taking far more than one’s fair share is a hallmark of capitalist societies (Sachs, 2012).

What is common to all these processes is that when we engage in social behavior, we are engaging in reciprocal, dynamic dances that are physiologically powerful and regulating, and stimulating different evolved algorithms in the mind. These “dances of our physiologies,” choreographed through our verbal and non-verbal expressions and communications, are regulated by different processing systems with different feature detectors. As we will note later, these dances are also socially contextualized and choreographed.

**FIGURE 3:**
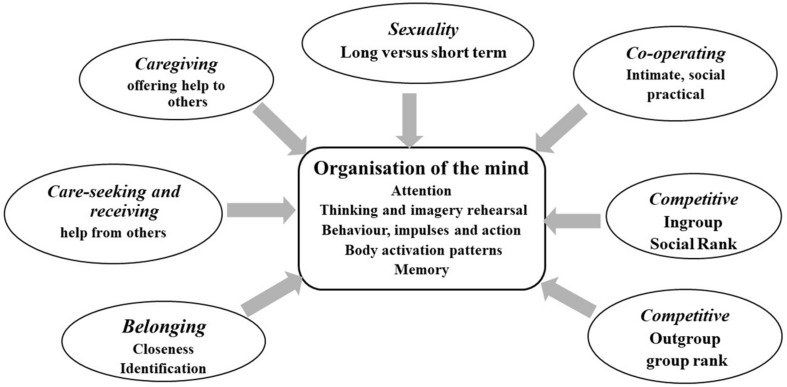
The social mentality organization of minds.

Figure 3 gives a brief overview of one way to conceptualize social mentalities. Each social mentality has specialist feature detectors to attend to different arrays of stimuli, picking up cues that are important and relevant to that particular motive and social mentality. For example, for sexual behavior we need specific *sexual detectors*, but for food, we need specific *food detectors.* We are not necessarily conscious of how our feature detectors are attuned to the environment (Bargh, 2017). Hence, each motivational system has its own algorithm with feature detectors that links into both specific and general physiological patterns. These are not Lego-like, but pertain to different combinations of different neurotransmitters, hormones, brain circuits and so on; different *patterns* of activation. For example, imagine your child is distressed and you are engaging caring behavior. Consider how your mind is patterning itself: what you are paying attention to, what you are rehearsing and imagining (what could the distress be?), what your impulses for actions are, what is happening in your body, to your heart rate, and are you remembering ways to be helpful? You would also be monitoring the degree to which what you are doing *is* addressing your child’s distress/need. If, on the other hand, you are the child, so you are the one distressed or needing and seeking care, then those processes will be different. For the moment by moment dance of your interactions to have an impact, there will be an attunement of interactions such that the caring behavior given is appropriate and the child physiologically responds in a way that is helpful to the child. Some of these co-regulating interactions of physiological systems are microseconds. Because of the way the human face has evolved, even an eye flash or slight smile can have quite a profound effect on an individual’s perception of friendliness or not. In fact, there is now a growing and fascinating literature on what is called interpersonal synchrony and this synchrony emerges out of the dances of communications and are physiologically synchronous. Child parent synchrony predicts altruistic behavior later in life (Cirelli et al., 2014).

Essentially then, social mentalities have co-evolved such that individuals can be receptive to the signals indicating a person’s motivation intent and then respond appropriately. This is the basis of role-sensitive empathy (Liotti and Gilbert, 2011). Some individuals can be very sensitive to say critical or hostile cues, but do not pick up particularly on emotional distress in others, whereas some people are the other way around. While social mentalities evolved for direct interactions, humans have also adapted them for use in wider groups including with strangers. The issue then becomes the degree to which a stranger is perceived as irrelevant, a threat, a potential resource, co-operator or requiring assistance.

These interactions are contextualized in social and cultural patterns that impact in complex ways on these interpersonal interactions and have a major influence on the manifestation of different social mentalities. At its simplest, in caring and sharing environments, individuals are orientated toward friendly, caring and sharing interactions, whereas in competitive environments the way we pay attention in our social milieu, how we reason, the way we wish to present ourselves to others, and the fear of how other people may see us, will be quite different (Gilbert, 2018). These concepts enable us to see how different social mentalities, that form the basis of interpersonal dances, and thereby physiological regulation, are culturally contextualized. Indeed, one of the major choreographers of the kinds of interpersonal dances created by our social environment in general is linked to the *resource competition strategies* that promote or demote either control and hold strategies or caring and sharing ones.

### Conflicts

A central driving process of evolution is conflict (Buss, 2019; Wilson, 2019; Workman et al., 2020). Right now, many species are in conflict with us because of how we use them for food, experiment on them, even deliberately altering their genes, and driving others to extinction. Whether it be it in parent–child, romantic partners, friendship hierarchies, and groups, it is the handling of *conflicts of interest and conflicts of desires* that is essential for how that relationship works. Here the quality of the relationship is important. For example, the way we care for our own children is quite different from how we care for the children of strangers. How we compete with our friends will be quite different to how we compete with those seen as competitors or enemies. Crucially then, the major sources of conflict throughout evolution are over *resources and resource availability and access*. Here, social contexts play a huge role. How conflicts of self-interest are dealt with in hunter-gatherer societies is very different from how they are dealt with in highly competitive, hierarchical, resource-rich societies such as ours (Ryan, 2019; Wilson, 2019). The ancient care and share versus control and hold strategies not only create *different contexts* for conflict but also indicate the means of resolving them. Put crudely, in caring and sharing environments it is to reach an equitable arrangement, in a hierarchical environment it is “the most powerful wins” and suppresses the needs of the subordinates.

Importantly too, in order to deal with conflict, different strategies have to turn on some motives, algorithms and emotions and turn off others. For example, if we want to go to war, then we have to turn off caring motivation and concern with the suffering toward these other human beings seen as a threat or the enemy we want to kill and maim. We can actually be pleasured and excited by their pain; have a rush of dopamine when we “take them out.” Clearly, in contrast to everyday forms of relating or with friends, if we are in a caring social mentality and saw that we had caused pain to another, that would be distressing. Generally speaking, much is done to stop us becoming aware of the intense misery we cause others in our wars, callous competitive behaviors, in sweatshops in far off countries and factory farms. Highly competitive motives tone down caring social mentalities (Piff et al., 2018; Basran et al., 2019). In addition, they inhibit empathy and being distress sensitive and steer us toward callousness, becoming insensitive to the suffering of others or even seeing them more as a resource to be exploited than to be nurtured or empathically connected to.

Even before Freud, we knew that the mind was riddled with conflict, with some motives and emotions able to suppress others. This is because different motives have different priorities and different physiological systems of activation (Ornstein, 1986; Huang and Bargh, 2014). Importantly then, motives and social mentalities can conflict and inhibit each other. This is important when we think about the social contexts that suppresses compassionate motivation and create brain states in interacting individuals that tone compassion down.

### Summary

The essence of the story so far then is that we are biologically designed with a whole suite of potential motives and social mentalities which organize our minds and bodies. We think these are all under our control, but in reality, they have been designed and evolved to be sensitive to the social contexts and niches we grow up in and have to function in Wilson (2019). While we are hard-wired for caring and sharing (Keltner, 2009; Ricard, 2015; Narvaez, 2017, 2020), we are also hard-wired with potentials to be a much nastier species. We now have insight that a major choreographer of these potentials that affect us all, right down to our epigenetic profiles and physiological organization, is resource allocation strategies and the management of the conflicts of interest. Just as we know we have phenotypic plasticity in the sense that any evolved motive can manifest in different ways, we know that cultures can have phenotypic variation too. While some cultures can support caring and sharing, others clearly do not (Black, 2016).

## Hunter-Gatherers and the Emergence of the Caring and Sharing Lifestyles

The caring social mentality (to provide care and support and be responsive to the needs of others) has been evolving over hundreds of millions of years, traceable in many ancient species and with the lineage of physiological processes increasingly well understood. The next piece in the puzzle, of how we became potentially one of the most compassionate species that have ever existed, and became highly regulated through our social relationships and need for connectedness, is the emergence of the hunter-gatherer group (Harari, 2014; Spikins, 2015; Lavi and Friesem, 2019). How the caring social mentality became recruited as a social focus and a basic cultural form for interactions, resulting in elevating sharing and caring relating, and offsetting earlier dominant male and aggressive hierarchies as ways of distributing and gaining control over resources, is one of our salient questions (Boehm, 1999; Lavi and Friesem, 2019). Not only that, but compassion was very much part of this with the archeological record showing that early humans were very concerned with the suffering of others and cared for the sick and injured (Spikins, 2015, 2017). It seems there were many factors that came together that shifted us into a caring sharing species and for the most part suppressing aggressive competitiveness. Below are some of them.

### Childcare

The way in which the caring social mentalities became a prominent form of social relating in humans occurred in various and curious ways. One of our human adaptions was upright walking, but this was to narrow the birth canal right at the time when the baby’s head was evolving to get bigger. Two things happened. One was that birth became dangerous and painful, and indeed hunter-gatherers had/have high mortality. Second, human infants require considerably longer parenting and socially interactive parenting that facilitates huge transformations during development. The way we adapted to these realities played a role in the shift toward our caring and sharing style of living. Narvaez (2017, 2020) has written extensively on how the social contexts of human parenting and provisions of parents to their children are very different from today (see also Hrdy, 2009; Music, 2014; Ryan, 2019). Although hunter-gatherers parenting styles differed, there is general agreement what the common characteristics were.

#### Care of the Mother and Alloparenting

Unlike any other animal, a woman trying to give birth by herself could easily run into problems. In parts of the world where medical services are scant, infant mortality can be tragically high, and indeed was high in hunter-gatherers. Birthing and subsequent caring, therefore, requires extensive support, commonly given by female relatives (Hrdy, 2009). Mothers would exist in these supportive networks of kin relatives before and after pregnancy.

Networks of women would also act as protectors and infants were passed around between grandparents, aunts and sisters, thus, the infants’ brain was responding to multiple social interactions; seeing many smiling faces and hearing kind voices. These would all be stimulating care focused physiologies (Porges, 2017). Throughout the child’s life many individuals would be a source of comfort and guidance. This is called alloparenting which is defined by parental functions (care, protection, provision of food, comfort play) as provided by those other than parents (Hrdy, 2009). Indeed, it is possible for children to seek out others in preference to their own parents if the parent relationship is not such a good one. Alloparenting is not unique to humans, but it was during our hunter gatherer stage that it became a highly adapted, even essential trait (Narvaez, 2017). Not only did it provide the infant/child with multiple interactions that facilitates complex social learning, a sense of social safeness and social engagement, but it provided resources to the parents particularly the mother, giving her time to pursue social activities and rest. A less stressed mother will have better interactions with her offspring than a stressed ‘coping alone’ mother. In essence the basis of our social brain would have been highly supported through the ecology’s of hunter gatherer alloarenting.

#### Contrast Alloparenting With Today

Many women today do not have access to these important kin/community supportive networks that would have been available 24/7. They are more likely to have midwives that visit them occasionally or deliver their children in impersonal hospitals by strangers. After birth if there is no close family nearby, women will lack extensive female support and may spend many hours alone waiting for partners to return from work; and not all partners are that supportive or adequate replacement for female kin networks. This is grossly abnormal and robs both infant and mother of important, multiple sources of support (providing both with a secure base and safe haven).

Mother and infant should be seen as a co-regulating couple who both require care in contextualized supported networks. Depression among mothers is much lower in societies that operate strong kin supportive networks (Hagen, 1999). To put it in another way, isolation and lack of caring support increases risk of depression. A depressed mother, coupled with a lack of alternative sources of care and stimulation, can have serious consequences for her maturing infant. To build a society focused on the common good *would prioritize childcare as fundamental to our societies* to cultivate prosocial minds. It is truly a tragedy that so many children grow up, trapped in homes with frightened, frightening and disengaged parents that will profoundly affect the maturation of their brains. COVID lock downs have made this *much worse*, the consequences of which will not be known for some years and probably never fully will.

#### Extensive Breastfeeding and Cohabiting

In hunter-gatherer societies, infant mortality was high, but those who survived childbirth would then experience extensive breastfeeding for up to 5 years. They would be carried – having skin-to-skin contact with the mother – and sleep with or close to the parents. The key issue is the caring physical contact that impacts physiologies (Mayseless, 2016).

#### The Importance of Touch

For many primates, touch, particularly in the form of grooming, has soothing effects (Dunbar, 2014). Indeed, touching massage impacts on oxytocin and reduces stress hormones (Morhenn et al., 2012). For the most part, close physical contact with mothers is common, but for humans, so too was contact with other members of the group (Hrdy, 2009). This meant a child had multiple sources of joy, physical comfort, and play. For infants, children, and adults, physical contact from trusted and loving others is one of the most important sources of soothing (Carter et al., 2017). Sadly, in modern society, teachers are warned off physical contact for comforting children for fear of prosecution – to the extent that they cannot even rub in protective sun cream or hug them when they are distressed. The fear of pedophilia is shocking and tragic. Western society has become almost touch averse. When my wife had our daughter Hannah 38 years ago (unfortunately by cesarean because of a breech) she would lie on the bed with Hannah on her chest. On a number of occasions, the nurse warned us not to do that because Hannah would “get used to it” and “become demanding.”

#### Paternal Behaviors

In birds, both parents often co-operate and take on the task of feeding and protecting. Not so with primates. For most primates, males play very little role in the care of offspring and do not know who theirs are. However, humans are a notable exception. Changes in the facial morphology of evolving humans suggest a trend toward feminization, and a changing role of testosterone, associated with increased caring and social affiliation behavior within groups (Cieri et al., 2014). Muller et al. (2009) compared two African groups showing that high and low parental investment groups differed in testosterone levels in predicted directions. Laboratory studies of human fathers have also shown complex relationships between oxytocin, testosterone and paternal caring (Weisman et al., 2014). Crucially the way testosterone affects male behavior and status seeking is linked to context. In some contexts, testosterone is linked to status by aggression, but in others, by altruistic caring (Sapolsky, 2017). Varela et al. (2018) found that skin to skin contact with their newborn babies significantly reduced stress hormones in fathers. While avian species share parental caring and therefore are likely to be mutually physiologically influenced by contact with their infants, this is not the case for primates and is probably a specific human adaptation. Indeed primate mothers will not allow any other to hold their infants and in this regard humans are unique (Hrdy, 2009).

One obvious implication is that human males evolved to interact with infants and children differently to other primates. Therefore men should be provided greater opportunity to care and interact with their children *and* other children *and* interact with supportive female networks, as would have been typical for many hunter-gather groups. Pair bonding in hunter-gather society is uncertain and males would often not know who their children were (Diogo, 2019; Ryan, 2019), but many showed high interest in and care for children, although the ecology influences this (Gilmore, 1990). Today, we are still exploring the important role fathers have on child maturation. The same goes for siblings, aunts, and uncles. The idea that a male should be absent for most of a child’s upbringing, returning home from an office tired and grumpy to an isolated mother and child, is a gross abnormality (Narvaez, 2020). For some fathers, COVID lockdown has given them new opportunities to interact with children and discover new ways of relating.

#### Lack of Harsh Punishment

Individuals are socialized according to the demands of the ecology. In dangerous ecologies, where men have to be relatively fearless, the social organization tends to be harsh, including to children (Gilmore, 1990). However, when in a benign environment, it is a different story. Many anthropologists believe that hunter-gatherers were averse to physically punishing or threatening children (Music, 2014; Narvaez, 2017, 2020; Ryan, 2019). Yet in post hunter-gatherer societies, and not very long ago, slave, wife and child-beating were allowed and legitimized and even promoted as a method for control (Gilbert, 2018). Even today, corporal punishment is still allowed in some countries. Caning was a regular practice in my boarding school of the 1960s.

#### Cooperation and Sharing and Caring

Cooperation makes hunting more efficient and this is true for various predators such as wolves, hyenas, monkeys and dolphins as well as humans. Humans extended cooperation into many domains of activity including cooking, building and making, and sharing of knowledge, which were facilitated through language. The most important driving force for language would be the sharing of information and knowledge. Indeed, *teaching*, which language facilitates extraordinary well, is phenomenally important to humans (Gilbert, 1989/2016). As noted below, status was not given to narcissistic self-accumulations, but altruistic sharing. What stands out too is the *enjoyment of shared activities* such as cooking and eating, which remains one of our greatest joys. In fact, hunter-gatherers worked far less than we do now and had happier lives (Ryan, 2019; Hunt, 2020).

In general, early human egalitarian behavior seems to have evolved under a number of conditions (Lavi and Friesem, 2019). First, rather small groups (150 or less that advantages reciprocity and other social processes; Dunbar, 2014). Second, a relative absence of storing, holding and controlling resources in favor of mobility and obtaining and sharing resource for immediate use (Lavi and Friesem, 2019; Ryan, 2019). Third, a sense of social belonging and social safeness was linked to displays of altruism, caring and sharing. Status was gained by being useful to others. Narcissistic, aggressive and exploitive forms of competitiveness were shunned. What became important was *competing for attractiveness* – to have a reputation as an altruist. This would make you attractive to others as a friend, ally, reciprocating partner and desired sexual mate. It is the domain of audience choice (Barkow, 1989; Gilbert, 1997, 2007). Fourth, as noted above, evolved changes to the birth canal from upright walking meant human birthing necessitated help and care through pregnancy and beyond (Hrdy, 2009). There was a significant shift in child-caring practises. Child care was highly interactive, non hostile, gave children a sense of interpersonal security and taught respectfulness, emotion regulation skills and the ‘joys’ of sharing.

It was the extensive kin networks that provided for children the opportunities to grow up with a secure base and safe haven, rather than being dependent on single parents that was crucial and different to other primates (Hrdy, 2009). Tragically today, many children are growing up bereft of these extended caring social contexts and indeed can be subjected to their polar opposites: neglect, hostility, criticism, and abuse (Lippard and Nemeroff, 2020). It is well known that many people who go on to have mental health problems and/or become power-seekers, anti-social or criminal often come from troubled and non-supportive or threatening backgrounds. Given that we have known about this for so long why, we might ask, have we allowed this *pandemic of poor caring* that generates millions of damaged minds to continue to operate? One reason (as we will see) is because control and hold strategies focus on individuals as “units unto themselves” who have to look after themselves and cannot turn to the community for support. It is a tragedy and a disaster because frankly, the world has been and is being run by quite damaged minds (Green, 2005; Lipman-Blumen, 2005; Owen, 2008, 2020; Black, 2016).

### Summing Up

Evidence suggests that humans adapted their physiological and cognitive systems for extended and extensive parent-infant caring (Hrdy, 2009) and these became a template for a range of social relationships, particularly in small groups. While caring and sharing in hunter-gatherer societies had a number of sources including conflict resolution and prevention, compassion as “the desire to intentionally help others in need and care for them” was certainly one of them (Spikins, 2015, 2017; Kessler, 2020). The basis of this is partly because the same hormones and patterns in the autonomic system underpin all forms of caring. Indeed, many argue that we are hard-wired for caring and sharing even, in certain contexts, with strangers (Keltner et al., 2014); that our basic nature is caring and sharing (Ricard, 2015). This was partly honed in hunter-gatherer groups where it works well and is a source of joy, social safeness and meaning. We can plot the genetics, epigenetics, physiologies, and brain patterns of caring and how caring became the rich textures for compassion. Indeed, if you think of how compassion can operate to steer us to risk our lives to save others, it is probably one of the most courageous and potentially wise of motives. Caring and sharing has a phenotypic profile that is choreographed in caring environments. In non-caring environments however the caring and sharing phenotypes are very different Here phenotypes orientate the brain toward the opposite of sharing caring and compassion that is callousness and the dark side. One note of caution should be added here. This is that we have been talking about the *biological* evolution of caring, but when the Buddhists talk about ‘compassion as our basic nature’ they are much more into the nature of consciousness itself and transpersonal realities rather than biological ones.

## Paradise Lost: Contest Competition and the Pursuit of Power

### The Development of the Terrorist State and Hierarchical Societies

Many researchers recognize that we have created social and ecological contexts that are very different to those in which we evolved, and in many ways we are quite mismatched to our new environments with unfortunate costs (Li et al., 2017). The more laidback and socially integrative lifestyle of the hunter gatherers can be very tragically contrasted with the 10,000 years or so which have not gone so well (Glover, 2012; Harari, 2014; Black, 2016). Armed with our amazing cognitive competencies and intelligences, that we honed in hunter-gatherer, caring and sharing environments, we turned these against ourselves to become the nastiest, most vicious, cruel and violent species to have ever existed on this planet. One of the great challenges for compassion *is not to be naive about humanity* and to recognize that we have a truly terrifying dark side. Hence, we need to keep in mind the challenges we face in turning our societies around and recognizing just where they have come from.

The basic story is relatively straight forward and one that many researchers agree on; it was the advent of agriculture that drove us a little crazy (Harari, 2014; Black, 2016; Gilbert, 2018). Our intelligence allowed us to recognize that rather than eat all our seeds, we could plant and cultivate them and could herd and cage animals. Resource availability spiraled but also, we became locked into areas of pasture, reliant on grown crops not gathered ones, letting go of the mobility and immediate use lifestyle. One of the most salient issues was that we went from an immediate use to a storage way of living and that changed the drive system as well as raised issues of who controlled what was stored. And if I can store things for myself then maybe I do not need to rely on reciprocation quite so much. So, agriculture changed our hunter-gathering lifestyles of relative immediate consumption, reciprocity and status via altruism into *stay put, create and store surplus and enable power hierarchies to control surplus. Humans themselves become trapped in completely new social ecologies* (Mann, 1986), which in many ways have driven us a little crazy and we do not really know how to get out of the trap our minds are now caught in Gilbert (2018). The work-time demands of agriculture and the social consequences of it almost certainly changed our bodies, brains and phenotypes. One of the major consequences was to promote the emergence of intense callousness and cruelty, as outlined below.

### Terrorism as Means of Control

As group size and resource value expanded, there was a re-emergence of aggressive, dominant (mostly) male hierarchies, the suppression of subordinates, and at times the vicious suppression of subordinate alliances against the hierarchy. The elites increasingly devised terror to suppress opposition internally and to terrorize enemies. Many leaders have left the defeated vanquished, tortured, executed, raped, and sold into slavery. The Geneva Convention is recent and sadly as conflicts in Syria and other places have shown rarely followed! Today we think that terrorism is relatively new, but throughout a lot of our history we have been ruled by terrorists and terrorism. Consider the Roman Empire. If you were born a slave, your life was horrendous and certain crimes were punishable by crucifixion, forced to fight in the “games” or torture. Around 72–71 BC Spartacus, who had been sold into slavery, instigated a slave revolt. Although the historical record is understandably hazy, it is generally believed that a particularly vicious and psychopathic Roman commander one Marcus Licinius Crassus defeated Spartacus and crucified all 6000 combatants lining the road with their crosses. He would often utilize a typical terror tactic within the army, such that soldiers seen as cowards or who had committed some other crimes would be beaten to death by their colleagues. Julius Caesar, another rather unpleasant character, instigated basic genocide throughout Europe. Indeed, the history of the Roman Empire is riddled with leaders and political schemers who were bad and mad. However, many European countries have been the same. Many Kings and Queens of England have been tyrants (Green, 2005). It was only a few hundred years ago that, you could be picked up off the streets and chain ganged into the Navy and if you committed a crime, you could be forced to make your own cat-o-nine tails that would be used to flay you, often to death, or Keelhauled (Rogers, 2007). Just two months earlier, you were with your family in Plymouth! There was also a time when you could be hung for simply stealing food. Over the centuries, *vast numbers* of people were hung for relatively minor crimes (Gilbert, 2018). The history of slavery is a vicious and callous one; perpetrated by minds that saw another human being as nothing other than a resource. Tragically, there are still many humans that view other humans like this. Today, we have discourses on colonialism and patriarchy, but these pale in comparison to human institutionalized terrorism, which up until recently has been endemic and in some states still exists; in governments and criminal gangs alike. There are still far too many governments with leaders who find it acceptable to lock up, torture and make political opponents disappear and the numbers are sometimes terrifying.

Many leaders have had quite serious mental health problems and personality disorders that have had major consequences for their leadership and what they enticed countries to do (Green, 2005). This is true for modern leaders as well (Freeman, 1991; Owen, 2008). Today too, there are powerful leaders showing signs of serious personality and mental health problems (Owen, 2020). Individuals who come from brutal backgrounds engage in control and hold strategies in very brutal ways and have changed the course of history and even the genetic complexion of humanity. Genghis Khan (1155–1227), who became the leader of the Mongol Empire was one such. He had a cruel and particularly brutal upbringing that filled him with rage and vengeance and would have left his phenotype for caring and sharing severely undeveloped. He became one of the most ruthless, vicious, sadistic leaders in history; responsible for the most horrendous deaths and rapes of many millions. His primary motivation was vengeance and absolute power so that no one could ever threaten or hurt him again. “Hurt people hurt people” as they say and he and many like him show that on a grand scale. However, as an evolutionary strategy, he was a success. He sired many children, quite possibly thousands by his many wives and others he raped. Such activity may well have affected gene frequencies in populations today. Consider one study by genetic researchers (Zerjal et al., 2003):

The Y chromosome of a single individual has spread rapidly and is now found in ∼8% of the males throughout a large part of Asia. Indeed, if our sample is representative, this chromosome will be present in about 16 million men, ∼0.5% of the world’s total. The available evidence suggests that it was carried by Genghis Khan. (p. 720)

Now, I am not a geneticist and this is an extremely complex area, but again, we need to take seriously the possibility that since the advent of agriculture and the rise of aggressive males (and their kin) who came to dominate resource holding strategies, certain alleles have been spreading through human populations. What, one wonders, has the history of wars done to our phenotypes given that so many with gentler temperaments, suited for hunter gatherer groups, were frequently slaughtered by invading armies. As just one example, for hundreds of years, the Romans regularly engaged in genocide wiping out many peaceful groups. Does this increase the frequency of potentially ruthless ambitious phenotypes getting control of power and then causing havoc, which they have throughout history and are now still doing (Wrangham and Peterson, 1996)? Groups that adopt control and hold strategies and competition for control are ideal breeding grounds for psychopathic tendencies (Black, 2016). We need to come to terms with the fact that there have been many leaders in history that have changed the world (and us) for the worst and still are.

We often draw our thoughts of terrorism quite narrowly but actually terrorism can be defined as the way power is used to frighten, intimidate, bully, hurt, even torture others to control them. Abusive parents can terrorise their children, abusive religions can terrorise their followers with threats of punishment in hell or other nasties; burning heretics etc. Terror using states allow leaders to imprison and at times torture political opponents. Stalin was an obvious recent example who did this on a huge scale, but tragically there are many states in the world today where leaders use terror tactics to control the population and quell opposition. The freedom to control and hold can create problems, but the freedom of free speech is essential to promote a compassionate world. Once again compassion has to address the dark side of humanity and recognise how closely it stalks us.

### Group Violence

Much has been written on group violence and the nature and origins of war (Black, 2016; Sidanius et al., 2017; Hobfoll, 2018). In many primate groups (but not Bonobos; Tan et al., 2017), aggressive clashes occur at the boundaries, so group on group violence is relatively endemic in primate groups where they meet each other. Humans have been different, to the extent that hunter-gatherers developed networks over large geographical areas. In addition, rather than being aggressive and war like, before the advent of agriculture, for the most part the evidence suggests that hunter-gatherers did not encounter each other that often and when they did preferred peaceful coexistence with each other (Fry and Söderberg, 2014). It used to be thought that we exterminated Neanderthals, but now it turns out we have got Neanderthal DNA (apparently 4% of mine is Neanderthal) meaning that we engaged them sexually and part of their demise was possibly climate change,

Again, many researchers suggest that it was expanding group size (facilitated by agriculture) that created large(r) tribes who in turn began to encounter one another and covet each other’s land and resources; or at least their dominant elites did. Group on group violence has been the source of some of the worst atrocities and most extraordinary cruelties, particularly in how the vanquished are treated (Black, 2016; Hobfoll, 2018). It is also the source of what may eventually extinguish humanity if we do not control it. Today, truly *vast* resources are spent on maintaining and building local and national armies, weapons, and weapon research (including the horrific prospect of robot armies), far more than on medicine. Some countries literally bankrupt their country in the pursuit of arms! Margaret Thatcher an archetypal ‘hold and control’ politician was a great advocate of the arms trade (Gilbert, 2018).

Over many years Sidanius, Pratto and colleagues (Sidanius et al., 2017) have been studying an orientation to resource distribution, which they called SDO. This measures the tendency to favor hierarchical and unequal rather than egalitarian forms of social organization within groups and between groups. Ho et al. (2012) offer a major overview of the important findings from this research tradition as well distinguishing two dimensions of SDO:

The dominance dimension is characterized by support for overt oppression and aggressive intergroup behaviors designed to maintain the subordination of one or more groups, whereas the anti-egalitarianism dimension entails a preference for intergroup inequalities that are maintained by an interrelated network of subtle hierarchy-enhancing ideologies and social policies. (p. 1004)

Ho et al. (2012) point out that SDO leaders in both politics and religion are very common, and tend to be socially divisive, and seek to privilege their own group. As leaders they gain support through two common tactics: one is to elevate the sense of threat from other groups and second is to promote a sense that one’s own group (be it based on ethnicity, religion, class, gender, etc.) is special and entitled to unequal access to resources and thus to exploit other groups for one’s own benefit. SDO is associated with the belief that it is okay to drop nuclear bombs on people and blame them for doing so (Slovic et al., 2020). Indeed, humans are well known to enjoy seeing others suffer if they are perceived to be the threat or “the enemy.”

### Callousness and Cruelty

In order to behave cruelly and callously to others, you have to either not develope, or at least not activate mechanisms in the brain such as concerned empathy. Indeed, competitiveness does exactly that (Piff et al., 2018). Hein et al. (2010) have shown clearly that when we see one of our colleagues experiencing pain, various brain areas linked to empathy light up. However, these areas do not light up in the same way when we see someone who is in an opposing group or team receiving pain. Sidanius et al. (2013) found that SDO was marked by reductions in empathy. If leaders want to turn off peoples caring and empathic orientation, all they need to do is to classify the other as a threat or an enemy and the brain will do the rest. Our preparedness to support the dark side of humanity is one of our biggest challenges (Hobfoll, 2018). Even the scriptwriters of Star Wars or Game of Thrones recognize these archetypal patterns are serious dangers to all of us if we do not control them. Currently, some political leaders and the right-wing media alliance fuel them rather than quell them (Sachs, 2012; Owen, 2020).

The story of power-seeking “control and hold,” not uncommonly fueled by leaders from troublesome backgrounds and at times serious mental health and personality disorders (Green, 2005; Black, 2016) is the (ongoing) story of tribes and empires crashing through the world leaving a havoc of tortured and mutilated bodies behind it. Less than 100 years ago, we had the Holocaust, and before that, in World War I an example not of a political leader but a scientific one. Fritz Haber was enthusiastic about the war and inventing mustard gas, knowing perfectly well the horrors of the death on a vast scale it would cause. However, it was not just the fact he invented mustard gas, it is the fact that so many were prepared to manufacture, deliver and spread it. It is not just the fact that the Nazis came up with Holocaust, it is the fact that so many were pulled into supporting it. Many took part in the denunciation, arrest and humiliation of Jewish people. Educated doctors and nurses experimented on children; many soldiers and even local people rounded up people and simply machine gunned them (Glover, 2012). Similar in many wars including Rwanda and the Balkans. So, unsurprisingly, around the world today many governments are corrupt, secretive, deceptive, manipulative, and yet gain support and orientate their henchmen in the police or army to threaten and suppress their populations. This is sometimes egged on by the sections of the media (Mulgan, 2006; Sachs, 2012). Indeed, as Gilligan (2011) reveals, some politicians are ‘more dangerous’ than others. Many right wing policies, which are basically invigorated control and hold strategies, tend to increase mental health difficulties, crime, social inequalities and social division and increase a sense of threat. So, it is not only the politician that is problem, but also the strategies they ignite to ripple through the groups they lead.

Consider two key themes in our entertainments, “protection and vengeance,” which justify our enjoyment of cruelty. A typical story is of bad guys doing horrible things to women and children or threatening to poison the world and then the good guys, from James Bond onward, rush in to wreak their vengeance and protection; the more horrible the deaths of the baddies the better, and everybody goes home happy. All these narratives are subtle, but constantly coaxing us into competitive social mentalities and group differentiations; constantly fueling simplistic ideas of concepts of good and bad people, them and us and bad people should be punished. Worst still is that not uncommonly so-called evil is presented as a disease; so for example, in Lord of the Rings, the evil ones always look very ill, sick, and ugly! The fact is, of course, that control and hold strategies seek the best, the most beautiful, the most luxurious not the most ugly; it is that kind of greed that is the problem. But it is a very useful (often non-conscious) tactic to manipulate our minds. We are not coaxed into understanding that we are all part of the human race pursuing strategies we never chose; that we are all struggling with the realities and the suffering and miseries of life; we are all hoping to be happy and to flourish; see our children flourish, grow and marry and not to have their bodies splattered against walls in bombing raids or legs blown off from land mines. Those leaders that get power because they have damaged minds that make them ruthlessly ambitious can entice us to continue to damage each other and be proud of it. We are constantly being misguided in understanding the nature of our own minds.

### Subservience

This means that understanding the emergence and appeal of toxic leaders is part of the human story (Lipman-Blumen, 2005; Owen, 2020). We also need to understand how a potentially caring and sharing primate like us, gets pulled into behaving in the most extraordinary and atrocious ways on a vast scale. Crucial, is how we become so compliant in our own oppressive cultures. We are a species of mixed phenotypic potentials and mentalities that can take control of the mind and make us heroes of resistance or highly subservient. The serious problem we have as a species is with our potential for callousness, cruelty and harmfulness. These have been the central focus of many spiritual traditions and scientific scrutiny (Baumeister, 1996; Staub, 2003, Zimbardo, 2008). Bandura (2012) suggests 12 possible reasons for our hostilities to others including vengeance, righteousness, protection, belief in a cause, justification and shifting responsibility. The contemplative traditions Jainism, Buddhism and others understood very clearly our potential for callousness and highlighted the importance of training the mind in forms of compassion to stand against it (Ricard, 2015). Concepts of reincarnation were partly instigated as threats against behaving harmfully in this life. For the most part, religions like Christianity also appeal to their followers to “love thy neighbor as thy self” and yet it was turned into a persecuting religion that was to see the Crusades, the Inquisition, and other wars in the name of Christ. Some of the tragedies of the Catholic Church include inducing fear in women over the right to choose their own birth control, and right-wing Christians cheering economic policies that promote deep inequality.

For the most part, western philosophical and psychological traditions have endorsed two basic views to explain the dark side of human nature. One is a kind of Hobbesian view that humans are out for themselves and will do anything to advance themselves and can only be constrained by an authoritarian state. Others argue that we are hard-wired and born to be (and need) caring and sharing and that it is combinations of culture and perhaps poor early life experiences that deviate us off that path (Keltner, 2009; Narvaez, 2017, 2020). There are two basic causes for that deviation. One is that we can be enticed into behaving harmfully because we are compliant and subservient to authority. In the 1960s, Milgram’s (1974) famous experiments enticed people to deliver painful and potentially dangerous shocks in learning experiments. He highlighted the human potential to be compliant and submissive in carrying out orders (Milgram, 1974). Zimbardo’s Stanford experiments at the same time implied that, give people a role (like a prison guard) and they will gradually adopt the (callous) behaviors of that role. Later, Zimbardo went onto explore a whole range of processes that facilitate good people doing *very* bad and cruel things that he labeled *the Lucifer effect* (Zimbardo, 2008). He was one of the investigators of some of the tortures that happened at Abu Ghraib during the Iraq war. Zimbardo (2008) and Sapolsky (2017) both highlight *the contextual and social conditions* that stimulate the worst in us and that we cannot understand humans as autonomous beings – a treasured but completely erroneous belief of the right-wing. We are an evolved species that adapts to its social niche for good and for bad. Having said that, it is also important to recognize that even in the Milgram’s experiments around 35% did not comply and the issue of rebellion is as important to study as the issue of compliance. Indeed, in psychotherapy, working on peoples’ ability to rebel against internalized authority or hostile others is essential (Gilbert and Irons, 2005).

Haslam and Reicher (2012), however, argued that compliance is complex because it is not just about fear of authority or passive subservience. It is also about identifying with values and goals and at times, being prepared to carry out harmful behavior. Indeed, people can become very enthusiastic about being harmful. There is a shopping list of reasons from compliance, protection-defense, coercion, greed, vengeance, personality, enjoyment, and physiological processes (Baumeister, 1996; Staub, 2003, Zimbardo, 2008). Consider that some religions have worshiped gods that required people to sacrifice their own children (The Aztecs). In 1978, Jim Jones, a famous cult leader, got more than 1000 people to kill themselves and their children. Consider what would entice or enable men to run at each other with swords and spears watching their friends around them being killed horribly? How many billions have died this way in agony? (Kelman and Hamilton, 1989; Shay, 2010). We can also consider that for 1000 years Chinese mothers broke the feet of their young daughters sentencing them to a life of pain. Still today, genital circumcision continues within communities around the world.

Not just in war, the dominant elites have operated terror regimes to maintain power. However, this has only been possible because it has been *accepted, even endorsed* by the subordinates and the oppressed themselves! In Europe, if you got convicted of certain crimes, you could be hung drawn and quartered for public spectacle. The public often did not object to this type of terror, rather it became entertainment. And there are forms of execution such as stoning, where the public are invited to take part! These historical process attest to the complex link between leaders and those who support them and this interaction is increasingly important to understand in right wing populism which can sow the seeds of harmful strife and conflict (Mols and Jetten, 2020).

## Gullibilty

Related to subservience is the issue of *gullibility* (Mercier, 2017). This is the degree to which people can be enticed to believe things that are not only untrue but are also against their own interests. While researchers believe that humans have capacities for identifying truth in messages (Mercier, 2017), it is also recognised that the modern media and ways in which leaders present themselves can easily overwhelm this competency. Some believe our ‘gullibility’ is one of the most serious challenges to democracy. To offer just two examples, some of President Trump’s supporters genuinely believed that the election had been stolen; others genuinely believe that the COVID-19 vaccine is either not needed or potentially dangerous. Part of the problem is the degree to which individuals identify with a social group, pick up the beliefs and concerns of that social group and reinforce them through sharing messages, for example on social media. To change ones view is therefore to change one’s group identity and sense of social belonging. We come again to the fact that humans do not make decisions purely on the basis of rationality but many other factors (Shu et al., 2019). It is therefore very easy to see how a deliberate false messaging of other people as dangerous, irresponsible or inferior can undermine compassion. The point is how we are complicit in the acceptance and peddling of false information. We are subservient to the distortions of our group.

### Competing for Attractiveness and Status: The Pros and Cons

One of our major transitions from a more aggressive primate past to hunter-gatherer care and share lifestyle was the movement of competition by aggression to competition by attraction (Barkow, 1989; Gilbert et al., 1995). Like so much in evolution, this has had some good effects, but also some downsides. Competition by attraction means one cannot simply impose ones will on others; one has to stimulate an audience to bestow status and share access to resources. The “group” decides what is attractive and worth giving status to. Indeed, it is now known that competing for status, with experiences of pride or shame, is a major common dimension of human social behavior (Gilbert, 2007; Durkee et al., 2019) and linked to reputation (Sznycer et al., 2016). Many on the right-wing argue that competition is important because it drives enterprise, and you cannot share resources unless you create them. While this is true, this confounds two quite distinct processes. First, over what and how people compete. Second, once created, if material resources do flow to winners, how should they be distributed? The control and hold mind sees competitiveness, mostly with regard to material resources and “the status of wealth,” not status via appreciation and gratitude, or altruistic reputation (Kasser, 2016). Wealth buys access to many privileged positions including opportunities to meet other wealthy elites, to partake of the fineries of life and to make those into an aspiration for the many. Altruism and non-holding of personal wealth as a status marker, that was so central to hunter-gatherers, has been reversed.

Competition is very important to humans and care and share strategies do not seek to suppress it but focus it. Central is how and what we compete for and how and what we do with the “winnings.” While some in the pharmacology industry are seeking to create the new COVID-19 vaccine in order to profit, many of the scientists working to create the vaccine are not motivated by the prospects of material resources (although they would not shun them if offered). Interviews with the scientists suggest that they are working from the sadness of the suffering, joy of being successful and the excitement and personal satisfaction of success; and of course status within that field. Volunteers who are subjecting themselves to vaccine research are not seeking material resources, but simply to be of value and to feel a sense of pride because of what they have contributed. The availability of potentially large sources of wealth has significantly changed the dynamics of human competition from our hunter-gatherer ancestry, which was mostly for social recognition, gratitude, appreciation, a sense of being valued and wanted in relationships of reciprocity, to self-focused control and hold and making that a status marker. Hence, individuals who can “stand on their own feet” and make money are regarded as higher status than those who cannot.

If you see the world as a constant competition for resources, then you can be “inferior” compared to others in your reference group; we rarely compare ourselves to people living in shanty towns but to other people like ourselves. Millionaires can be driven to compete because others have got more millions than they have; this is competition out of *envy and fear of inferiority* (Basran et al., 2019). Second, when it comes to taxation, wealthy folk (understandably) seek to defend their position and resources and pay the least including trying to hide what society requires them to pay (Jetten et al., 2020). So in creating a world of riches, rather than making us feel safer, it has a paradoxical effect of making us more threat focused, socially comparing to our local reference groups, and as a result, callous to the less well off (Piff et al., 2018; Basran et al., 2019).

Competing for status has other problems. Shame and stigma are the underside of competing for attractiveness (Gilbert, 1998, 2007). Status competition is also linked to the *suppression of competitors* which is the function of shaming and denigrating (a major problem in social media). When our minds are organized by the competitive mentality, the drive for fame and the fear of shame textures our minds, leaving us more disconnected than ever. Buss and Dedden (1990) refer to this process as the derogation of competitors and highlighted the fact that shaming and stigmatizing focus on different attributes in men and women. One of the ways in which class and caste hierarchies are maintained is through the process of down rank shaming and stigmatizing.

The issue of competing by attractiveness can also create extraordinary stigmatizing hierarchies. For example, countries with a caste system assign groups to be as untouchables and excluded from the benefits the higher castes. Regardless of the origins of the Indian caste in the 15th century, it was the British Raj system, which intensified these divisions because it facilitated their capacity to divide and rule, as well as offering favored positions to certain individuals from the higher castes. All kinds of class system carry with it judgments of “attractiveness” and “social worth’ – with those who are in the higher classes believing they are worth more than those in the lower. SDO which would certainly involve the desire to maintain class and cast distinctions goes with lack of empathy for those below; or as Sidanius et al. (2013) call in their paper “you are inferior and not worth our concern.”

Another dark side of competing for attractiveness is the issue of honor and the acclaimed right to defend one’s honor with violence. Cohen et al. (1998) show that in different countries and even different states in America, people can take different views to the use of violence to defend honor, particularly in men over women (Vandello and Cohen, 2003). More tragically is how some communities have created systems of honor, where parents are able to kill their own daughters if they have in some way dishonored the family (Sanghera, 2009). Consider what must be happening in one’s brain to want to do that? Face saving and vengeance over social shaming can be tragic. And groups can also inflict horrific deaths, stoning of women being one.

## Control and Hold. the Pursuit of Power and the The Dark Side

While some competitive behaviors can be beneficial, unregulated competitive behavior that seeks to suppress others can have a terrible dark side. Indeed, it *is the use of power* to suppress and exploit or act out the traumas of childhood (as people such as Genghis Khan, Hitler, Stalin, and co have) rather than to help, that is the issue. In a hunter-gatherer group, aggressively competing for power would have been shamed and even have got you killed. However, that is exactly the survival and reproductive strategies followed by most mammalian groups. The distribution of resources emerges from contest (rather than scramble) competition, especially but not only over sexual/reproductive opportunities where usually the strongest will try and inhibit subordinates from mating. Hierarchies of this type exist for both males and females (Sapolsky, 2017). While males are more aggressive, and can evolve traits that are designed for intrasexual fighting (e.g., size, consider the thick skulls of the long-horned mountain goat), female primates will certainly try to inhibit subordinates in their resource seeking behaviors (Gilbert and Basran, 2019).

Indeed, many researchers have drawn attention to the way contest competition has generated a range of motives and psychological processes. For example, Price (1972) and Gilbert (1989/2016, 1992/2016, 2020b), and Sloman and Gilbert (2000) explored how the psychology of social competition, linked to social comparison, dominant-subordinate interplay, and shame proneness underpin vulnerabilities to a range of mental health and anti-social problems. Johnson et al. (2012) suggest we have a “dominance behavior system” that can be detected in social behavior, peoples’ psychological focus and physiological organization. They suggest that:

Despite the variability in the terminology used by different authors and in different fields of study, there is agreement regarding the existence of such a system in all mammals and regarding the ultimate goal this system serves: namely, *control over social and material resources* or what we call power. (p. 693, italics added)

Eisler and Fry (2019) suggest similar, but with a different focus on what they call *a domination system*, with a four-component system that links evolved motive with cultural practices. First is rigid top-down impositions of power. Second is the tendency to see one group as superior to another and fair game for exploitation, suppression or even removal. This is close to the concept of SDO, which again is about control and hold strategies for resource competition (Sidanius et al., 2017). Third is the cultural acceptance of the means to maintain the social hierarchy, typically through power plays, acceptance of the elites and the use of violence and the threat of violence. Indeed, as noted, humans have been very prepared to watch and endorse the most horrific cruelty of individuals deemed undesirable via crucifixion, burning, flogging, stoning, and so forth (Gilbert, 2018). The crucial issue, however, is not power itself, but how power is gained and how it is used and in particular how it is used to deal with inherent conflicts and issues of resource distribution (Mulgan, 2006).

The creation of hierarchies, especially power hierarchies, impacts on how subordinates and the powerful relate to each other. Essentially if you are pursuing power and going up the ranks, you need a mind that is orientated to facilitate that behavior. This is not a mind you choose. This is a mind evolution and culture has built for you. Hence, it will tend to orientate your attention and thinking in a certain kind of way and it will also have an impact on your use of empathy and desires for sharing (Decety and Ickes, 2009). For example, Keltner et al. (2003) offered a major review of the psychology of power. To quote from their clear summary, they note that:

Power is associated with (a) positive affect, (b) attention to rewards and to features of others that satisfy personal goals, (c) automatic information processing and snap judgments, and (d) disinhibited social behavior. In contrast, reduced power is associated with (a) negative affect, (b) attention to threat and punishment, to others’ interests, and to those features of the self that are relevant to others’ goals, (c) controlled information processing and deliberative reasoning, and (d) inhibited social behavior. (p. 265)

These issues are closely related to a personality dimension called *narcissism*. Although somewhat controversial, there is a general view that these individuals are dulled down when it comes to compassion and empathy for others and very activated when it comes to the competitive social mentality, self-promotion, self-absorption, sense of entitlement, and willingness to exploit others for one’s own benefit (Zeigler-Hill et al., 2019). Basran et al. (2019) also found that people who endorsed hyper-competitive, narcissistic and ruthless self ambitious views also endorsed anti compassionate attitudes and resistance to being helpful to others. While we can understand these as individual and personality issues, it is important to also see that cultures stimulate these phenotypic potentials quite automatically; these are not chosen. In addition, one can see the theme of SDO as a form of social, cultural narcissism.

### On the Nature of Threat and the Fear of Losing

Different strategies will recognize different threats to their enactments. The threats in agricultural-based, high resource-storing environments, with individuals pursuing competitive resource control and hold strategies are quite different from the threats in hunter-gatherer societies. Their threats are to their immediate survival and for all members to pull their weight and share their resources (Lavi and Friesem, 2019). These are also reflected very much in how threats are represented in different political philosophies. There is increasing evidence for example that right-wing individuals, who tend to endorse the control and hold strategies, are threat sensitive to themes such as national identity and the freedom to *control and hold* personal resources (Perry et al., 2013). Fear and anger are more easily triggered if one is concerned and vigilant that others are trying to take one’s resources away, compared to believing others will share theirs with you.

So, these strategies drive very different attention and defensive emotions. The wealthy may fear the claim of subordinates taking their resources in demands for equality, whereas the subordinates can feel impoverished, let down, excluded and neglected and want their share of the pie (Gilligan, 2011). There is now considerable evidence that in competitive environments that create disparities, there emerges the fear of failing, falling and losing. (Jetten et al., 2020). Those who are especially competitive, ruthlessly ambitious, and narcissistic have a high fear of inferiority and losing out (Basran et al., 2019). In children and young people, perfectionism and the fear of failure has intensified under neoliberalism, driving mental health problems (Twenge and Campbell, 2009; Twenge et al., 2014; Curran and Hill, 2019; Becker et al., 2021). As reviewed elsewhere, this is associated with self-preoccupation, self-criticism, and less interest in the welfare of others (Gilbert et al., 2020). In competitive societies, when the future looks less rosy or opportunities less viable rather than seeing this as a reason to pull together, it can intensify control and hold thinking. Jetten et al. (2020) highlight this as one of the reasons for what is called the wealth paradox whereby the wealthier individuals become, the less they want to be helpful to others (Van Kleef et al., 2008; Piff et al., 2018).

Note too, that poverty prevents talent from coming forward and competing for social share. The wealthy can pay for education and advantageous social networks (Murray and Frijters, 2017) in ways the poor cannot (Galbraith, 1993). In addition, poverty creates the social divisions for some forms of criminality and mental health problems, hence maintaining low subordinate “power.” So these *control and hold* and *care and share* strategies tune attention, motives, emotions, ways of thinking and physiologies in quite different ways. The strategy of control and hold requires individuals to believe in it, thereby support the importance of competitiveness, hierarchy, inequality and self-determination and attend to and defend against threats to it. If the strategy cannot create minds that are patterned that way, then it would gradually be reduced or be suppressed as a meme in the population. The strategies for care and share will try to orientate minds in very different ways.

### The Problems in Lack of Regulation of Exploitative and Cheating Hierarchies

While individuals can be trained for compassion and cultivate compassion, social and global compassion involves the recognition that human behaviour also needs to be regulated through social contexts and sanctions. We need to find compassionate ways to regulate cheaters, exploiters, power seekers and so forth; unregulated they can do and have done immense harm (Black, 2016; Murray and Frijters, 2017; Sidanius et al., 2017; Hickle, 2018). As noted above, Boehm (1999, 2000) argues that a shift into egalitarian ways of living might partly come in the ways alliances between the more subordinate individuals could depose bullies.

I made the case that humans can remain egalitarian only if they consciously suppress innate tendencies that otherwise would make for a pronounced social dominance hierarchy. In effect, it is necessary for a large power-coalition (the rank and file of a band) to dominate the group’s would-be “bullies” if egalitarianism is to prevail – otherwise, the group will become hierarchical with marked status differences and strong leadership.

On this basis, it can be argued that humans are innately disposed to despotism in Vehrencamp’s ethological sense of the word. My point is that humans are not just naturally egalitarian: if we wish to keep social hierarchy at a low level, we must act as intentional groups that vigilantly curtail alpha-type behaviors. This curtailment is accomplished through the cultural agency of social sanctioning …., so political egalitarianism is the product of morality. (p. 84)

Further support for this idea came in a strange and unexpected way. In the mid-1980s, a group of baboons that Sapolsky and his team (Sapolsky and Share, 2004) had been studying were affected by tuberculosis that, for various reasons, took out the most aggressive dominant males leaving a cohort of mostly females and less aggressive subordinates. The group settled into a very different pattern of low aggression, relaxed, caring, and sharing; what Chance (1988) might call a “hedonic mode.” Since males leave their group and new males enter, it is fascinating that a decade later, the same relaxed style persisted: incoming males were learning and co-creating a social context for more relaxed relating.

Gintis et al. (2003) also indicated that what they call “strong forms of reciprocity” can evolve under these contexts – but it will be associated with punishment for cheaters (Boehm, 2000). Hence, both the regulation of exploitative, hierarchy-orientated individuals and the regulation of reciprocal altruism probably arose together and both of them involved punishing those who would exploit or take advantage of others in a group. Interestingly, many commentators have pointed out that today this now benefits the elites: they punish the less well-off if they are deemed to exploit “benefits,” yet they are allowed to get away with extraordinary levels of tax avoidance and the harmful exploitation of the environment (MacLean, 2017; Murray and Frijters, 2017; Hickle, 2018). It is well known that unregulated competition invites cheating. Consider any major sport, particularly when there is a lot of money involved, the prevalence of cheating increases and the need for refereeing and policing is essential. Cheating can take the form of using drugs to promote performance or finding ways to undermine the opposition. In business too, unregulated competition enables multinationals to hide vast sums from taxation; some believe that there is more unpaid tax than actual paid tax. Some car companies have been discovered to be cheating on the information on how polluting their cars are. Many companies are not known for their ethics.

Part of the problem is that although Boehm (1999) is correct, we are not sure how to do this in big groups. In our mega groups of strangers, as we become wealthier, we begin to endorse ideas that we deserve our wealth. Be it film stars with talents to act, footballers to kick balls or bankers to move monies around stock markets, their multi-million pound salaries are deemed acceptable and deserved. The environment of control and hold has to generate minds that think like this otherwise as a strategy it would quickly fail; people could be burdened with guilt. In addition, we have a mind such that giving up resources as in taxation, feels as if things are being stolen from us; and our brains are particularly attuned to seeing the loss of resource as a threat (Jetten et al., 2020). Ensuring egalitarianism in a hunter-gatherer group, where one is in direct contact with the beneficiaries, one experiences reciprocity and gains appreciation, is very different from paying taxes, especially when we do not agree with how those taxes should be spent (e.g., on nuclear weapons). We are constantly battling with the nature of the evolved human brain and unless we take this on board, moving forward will be difficult.

### Economics

We need new economic models and science, not just to study the forces of demand and supply. We need to figure out how to create economic systems that directly *impact the brain and the mind* for the common good that involves more caring and sharing to address inequalities. We know that economic disparities and in particular poverty have major impacts on brain development, function and epigenetics (Shonkoff, 2011) and mental health (Becker et al., 2021). The issue is going to be whether we will turn a blind eye to the problems of *control and hold* and simply continue to allow these strategies freedom to express and spread themselves in populations (like a virus) or learn how to inhibit them in favor of *caring and sharing* ways of living. Doing so would mean coming to terms with evolved strategies that have been battling it out in our minds and groups and genes for millions of years. Unregulated capitalistic models that seek to create free markets are utilizing strategies *for resource competition* where the strongest or most able gain access to resources and can also put in place strategies for the inhibition of competitors (and subordinates). This has seen elites throughout history exerting extraordinarily callous and cruel regimes of oppression against the poor and still do (Perry et al., 2013; Black, 2016; Murray and Frijters, 2017; Sidanius et al., 2017). Resource competition is at the root of all this.

Many western philosophers have also taken very different views about the nature of rights to accumulate power and resources and exploit them for personal benefit. John Locke (1632–1704) believed “in the right” to accumulate and exploit. Some of his ideas landed in the American constitution, shaped by the fight to rid themselves of British external control. Arthur Schopenhauer (1788–1860) on the other hand was very taken with Buddhist philosophy. Regarded as a pessimist, he saw the world as an unpleasant place full of suffering and for which compassion was the main antidote, including the basis for economics. For him, accumulation could not be a right, because my accumulation is your loss. Such debates based on whether people should *hold and control* and be allowed to accumulate or whether people should *care and share* orchestrated by the state have been given many new twists and turns with both sides claiming that their strategy improves the common good [as between Nozick (1989) and Rawls (1999)].

Recently, Mark Carney, ex head of the Bank of England gave the 2020 BBC Reith lectures called *how we get what we value*. His basic premise is that we have increasingly fallen into a world where things are decided on the basis of financial value rather than human value or that which will sustains us. Many of the crises we face, including climate crisis, cannot be solved by market economies focused on financial value. He notes that businesses are now beginning to think about sustainability and also that profit should be ‘with purpose’ not for its own sake. These are fascinating and important discourses arising within the business world. However, economics tends to make the assumption that each individual’s brain is pretty similar without accounting for individual variation. One problem with these discourses then is that they tend to be detached from the processes of how economic and social conditions shape the kinds of brains we have – right down to our epigenetic profiles. It is also important to consider that different economic systems and cultures privilege different personalities as well. Competitive cultures are fertile grounds for narcissistic and psychopathic personalities to prosper and gain power. Not only do they gain control of businesses and the media, but they then create social discourses that entice many into the control and hold way of living. We are definitely not the masters of our own minds despite the illusions of being so (Bargh, 2017).

### Turning Humans (and Animals) Into Resources

One of the mental processes *of control and hold* is to change our relationship with nature, other species and each other. The basic view of the first book of the Bible – Genesis – is that God created the world for humans *to use*. And use them we have. Our cruelty to animals is shocking both in terms of our factory farming and extensive, unnecessary and environmentally harmful use of them for food, and our whaling, hunting these highly sentient beings almost to extinction.

As for turning each other into resources, Genesis tells us that Eve was partly created for the benefit of Adam. This mindset is also captured in psychological concepts as *the objectification of others*. Many of the major problems that humans have with each other are precisely because we treat “the other” as a resource to fulfil our wants and needs or as a threat. Slavery is an obvious, horrific example. In fact, all the major civilizations are built on slavery (Black, 2016). The Vikings and Romans had a flourishing slave trade, as did the Chinese who used them to build their walls, and the Egyptians who used them to build pyramids. In all these societies, little attention was given to the plight or suffering of slaves or the less fortunate because the empathy and caring systems in the brain, necessary for that, are switched off in favor of control and hold shaping phenotypes. Again and again, we see that it is the culture that is regulating the brain and its phenotypes.

Consider objectification in sexuality. In a famous chapter called *The Man Who Mistook His Wife for a Chattel*, Wilson and Daly (1992) explore how males can *treat females like property* and a usable resource. Anything people desire, like sex, can become an exploitable resource. However, with the nature of human sexuality (it can last much longer than most primates engage with it) the enjoyment of sexuality, the bonding and friendship of sexuality, all point to human sexuality being different in hunter-gatherer societies (Diogo, 2019). Women were not simply resources, but had egalitarian choices. Bonobos too are matriarchal and use sexual contact quite differently, not as a resource, but as a way of dealing with conflicts and social bonding. Change the culture and you change these dispositions.

Another area where the *objectification of natural resources* has been a disaster is in land use. One of the effects of the emergence of hierarchies was that people sought to “lay claim to own” the land, which over time would lead to the policy of enclosure – forcing people off their lands and prohibiting access or use (Mann, 1986; Hickle, 2018). Land ownership not only came with prohibiting access and establishing monitoring patrols, but also poaching became a hanging offense. Such punishments, instigated by the elites to protect their interests, left the poor no means to stop or resist them. The idea that people should be entitled to control and hold and do whatever they want with their resources stretches back many hundreds of years. As a consequence, natural resources (from mines to forests to fisheries) have now been claimed as the property of the elites and exploited in very damaging ways. Hickle (2018) says that

…. Since the 1950s there has been an extraordinary increase in global GDP (often referred to as the “Great Acceleration”), but this growth in “private riches” has come at the expense of an extraordinary depletion of the living world, given the tight coupling between GDP and material and energy throughput. The majority of the planet’s tropical forests have been destroyed, agricultural soils are largely degraded, rates of species extinction are now 1000 times faster than the background rate prior to the Industrial Revolution, while CO_2_ emissions have caused climate change and ocean acidification, destabilizing terrestrial and marine ecosystems and threatening food chains. This is the ultimate price of the longstanding plunder of “free” value from nature. And by destabilizing the biosphere on which human life depends, it becomes clear that the greatest public wealth of all – the integrity of the planetary biosphere – has been sacrificed for the sake of private riches. (p. 55)

### The Tragedies of “Labor as a Resource”

There are other serious anomalies too. Individuals in hunter-gatherer societies had to dedicate time to the processes of living-surviving such as hunting, gathering, cooking, preparing shelters and rudimentary clothes and so forth. Evidence from studies of bone density suggests that hunter-gathers were very mobile and active. They were in direct contact with “nature,” the products of their labor, and the process of their labor was social. The activities of hunter-gathering built strong social ties. The effects of hold and control strategies shattered that into highly specialized divisions of labor where many individuals were turned into salves and servants or left with soul-destroying jobs simply to maintain some kind of hold on life. The industrial revolution may have marked progress in technology, but did exactly the opposite in terms of human dignity, happiness and social fairness. Although many commentators like Marx and Engels had highlighted the misery of the working classes, today we are beginning to wonder why we esteemed those entrepreneurs that created such dark Satanic Mills, the coal mines and factories and who drove colonial exploitation which devoured the human spirit and spiraled it into such a miserable existence. Yet, we have inherited these same forces that trap us, so that now we have become what Madeline Bunting (2004) calls *willing slaves*. We cannot easily survive without being part of a production process that eats deeply into our lives. And many have a need-hate relationship with it in the sense that we need to work in the factories or wherever but we hate having to spend such a large percentage of our lives doing so. Yet without that opportunity we would not have the resources to be free of poverty. Everyday we have to spend hours in traffic jams and doing things we do not want to do. Some feel so disengaged from the joys of living, sacrificed on the altar of routinized working, that we live in varying states of frustrative, envious and helpless anger, and emotionally dissociated states (Bunting, 2004). Many dream of winning lotteries that will release them.

To distribute resources and create opportunities to contribute to a sharing and caring world has never been the desire of competitive, neoliberal politics or the right-wing (Gilligan, 2011; Sachs, 2012). It is competitive self-interest *to control and hold* as much of the resources as one can. Resource control buys freedom from hunger, comforts, pleasures, and medical care; ways to make the world more enjoyable and safe for oneself and kin (Galbraith, 1993). It is sold under the banner of liberty and freedom to secure as much personal wealth as we so wish, but this is exactly what would have been shamed in hunter-gatherers! And commonly, it comes with shedding of responsibility for others and extraordinary justifications for exceptional wealth (Galbraith, 1993). Indeed, as history shows so clearly this so-called “freedom and liberty” also involves freedom and liberty to exploit and use others as a resource if you possibly can. This is why we have had colonialism, slavery and companies that drive down wages and can behave in psychopathic ways (Bakan, 2012; Black, 2016). There is a difference in freedom from and freedom to. In addition, the pursuit of wealth is addictive and the more one has, the more one wants (James, 2008; Piff et al., 2018). Without insight into how these evolved strategies can “run” our minds and be addressed at a deep, archetypal and evolutionary level, the desire for global compassion may remain simply a wishful desire (Ekman and Ekman, 2017; Gilbert, 2018).

COVID-19 has reminded us of two essential life realities: Firstly that this life is incredibly short and fragile and in any moment we can suffer sudden changes in our ecologies, the flushing through of a major virus or other diseases that can decimate whole populations (human and non-human alike), and the vicious eruptions of tribal violence. Yet secondly, what we have always intuitively understood is that when people are given the chance to contribute, and the closer we get to a hunter-gatherer type social group, the more caring and sharing people become, the happier and healthier they become. The great challenge for us is to now support that basic motivation to be a contributor (rather than a taker) in a caring and sharing world, even when the recipients of our help may never be known to us (Loewenstein and Small, 2007). That starts with an understanding that while we all want some comforts, after a certain point, wealth does not add to happiness or life’s meaning. Hence, we can come to see the value of letting go of a need to pursue excessive accumulation and replace it with a desire for contributing and personal contentment (Cordaro et al., 2016). Indeed, various commentators have highlighted the importance that contentment, rather than striving for more, is a way to long-term happiness for all (Naish, 2008). Contentment does not drive capitalist economics and if it started to flourish, marketeers would try to find ways to undermine it to keep us on wanting. Our challenge is how to find a way of creating a world where people are enthusiastic about developing and using their skills and talents and are facilitated in doing so, but in ways that support the common good. One of the most extraordinary things about the Internet is the number of individuals who are making available all kinds of educational material from how to grow vegetables, fix cars, play the piano, learn a language, and cook fairy cakes. They do this purely for the joy of sharing and wanting other people to benefit from their knowledge. It is these motivational systems we need to find ways to harness because there are many entrepreneurs who simply love inventing and creating and do not need multi-million pound yachts to do it (Toynbee and Walker, 2009). We need to find ways to help as many as possible find the love of creativity, the joy of contribution rather than the love of money.

## The Mind Games of the Ancient Strategies

These kinds of strategies need to create receptive minds. As noted above, this is because social motives co-evolved. Individuals who expressed caring will need individuals who are responsive to caring. Individuals pursuing sharing and cooperation need to stimulate those strategies in the minds of others and suppress control and hold strategies. Individuals who express dominant behavior need individuals with evolved mechanisms for submitting themselves, who are willingly to do so rather than fight back. Control and hold strategies need to create a climate where a number of individuals have those strategies stimulated and engage them, and as indicated above, stimulate fear for one’s own livelihoods. Hence, these strategies will (simply because of how they are built, not because of any conscious intent) try to stimulate minds around them to create an information “flow between minds” that supports them. This information flow does not need to be accurate, it just has to ‘push the right buttons’ in the minds of others. For those strategies to work, they will also have to organize the human brain in a certain pattern. For example, hold and control strategies also have to cover their tracks, so they switch off empathy and guilt, so people will not experience emotions that might make them rethink their actions. Just as Romans would not have found anything unusual about using slaves as they did or going to the gladiatorial games, so the industrialists of previous centuries would not have thought of their colonial behavior as callous exploitation; and in fact, some of them were esteemed back home for their behavior. The British exploits of India are but one example. Indeed, the past appalling behavior of the Europeans in the Americas and the abject suffering, misery, and destruction of whole cultures all display examples of when people have viewed their behavior as worthy of esteem rather than condemnation. The culture is crucial in the acceptance and adoption of the strategies of resource control. Looked at this way, it is not power hierarchies that is the issue, it is how power is used in the pursuit of resources and who and what is *made into a resource* (Lee-Choi and Bargh, 2001).

## Summary of Comparisons

Table 1 brings together some of the themes addressed above in terms of how different strategies organize the brain. Of course, minds do not operate Lego-like systems but complex networks of dissipated circuits that produce patterns of electrochemical activity underpinning motives and behaviors. Hence, it is preferable to think of these as *multiple dimensions* that we can all move along to some degree according to context and life stresses. This is why it is useful to practise mindfulness and notice (without criticism) when these strategies are operating through oneself (Gilbert, 2009, Gilbert and Choden, 2013; Ricard, 2015).

**TABLE 1:** Differentiating biopsychosocial processes according to evolved resource regulation strategies Distinguishing minds.

Hunter gatherers –pre-agriculture care and share	Modern post agriculture control and hold
•Egalitarianism •Inhibit strong competitiveness •Support respectfulness •Supports caring empathy •Suppress narcissism •Strong group bonds •Group parenting •Expansive play •Relatively open sexuality •Child-focused •*Sharing and caring*	•Hierarchical •Heighten competitiveness •Allows exploitation •Limits caring empathy •Reward and promote narcissism •Weaker groups bonds (the elites) •Limited parenting •Constrained play •Controlled sexuality •Adult focused •*Controlling and holding*

**FIGURE 4:**
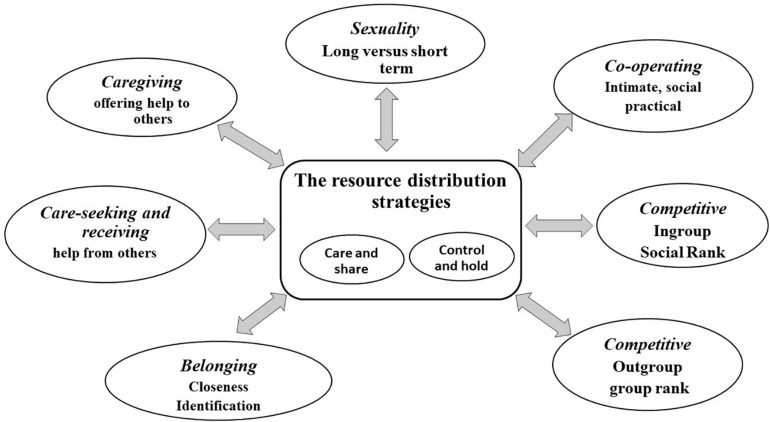
Resource distribution strategies in social mentalities.

Figure 4 illustrates how social and culturally endorsed and promoted resource distribution strategies impact on the social mentalities, including their physiological profiles and phenotypic forms. For example, consider caring behavior in hunter-gatherer groups, which were (commonly) child-focused compared to the factory workers of the 18th century. The variation in these two environments would have a huge impact on the maturation of a child’s brain and epigenetic profiles. Consider how competition and the propensity to develop highly narcissistic self-focused orientations emerge in competitive cultures compared to caring and sharing ones. Again, the social and ecological environments will have major impact on the phenotypes of competitive and caring behavior. This is important because these phenotypes will be regulating motives and competencies, turning some up and others down (e.g., compassion and empathy).

Figure 4 indicates an interaction between what culture and political system privilege, the regulation of individual minds and then how those minds support the accepted resource distribution strategies.

## Creating Compassionate Worlds: the Hope and Disappointment

Amongst the wars and atrocities, the state use of punishment, torture and (at times truly horrific forms of) execution, and the abject poverty and misery of the poor, humans have also tried to find better ways to live together (Sachs, 2012; Wilson, 2019; Biglan, 2020). Sometimes it is two steps forward and one step back, but nonetheless, many might agree that we would prefer to live now, with our modern medicine, comforts, and greater freedoms than even just a few centuries ago. Political landmarks in the West might be the ending of industrialized slavery, introduction of child and labor employment laws, education, expanding democracy votes for women, freedoms of speech, and freedom in sexuality. Tragically World War II reminded us what a vicious and dangerous species we are with the Holocaust, the (fire) bombing of cities and the dropping of nuclear weapons along with the typical viciousness of local killings, tortures, and mass rapes (Glover, 2012). Surely, we can do better than this.

Concerns to create fairer and more humane societies are of course many centuries old. In the modern era, before World War II, British Prime Minister Lloyd George championed a range of social welfare policies and later American President Franklin Roosevelt set up the new deal focusing on the three Rs: relief from unemployment, recovery of an economy, and reform of the financial system. Wilson (2019) notes that the motives for such might have been mixed; partly compassionate but also concern with increasing social unrest from the poorest sections of society that threatened the welfare of the elites and these efforts were needed to soothe them. Germany, on the other hand, lumbered with the economic reparations from the Treaty of Versailles, signed on the June 28, 1919. This caused such unemployment and social unrest that it became the breeding ground of anger and revenge and support for one of the world’s most destructive tyrants and the rise of fascism. To this day, self-identification with a group that feels threatened or excluded can be problematic and a lever for political manipulation (Sidanius et al., 2017). After the war, there was the Marshall Plan to rebuild Europe to try to avoid the same mistakes and for opportunities for business. Never humiliate a defeated enemy. To show some compassion *and* develop business with them is a useful idea to prevent subsequent conflict; sad that so many conflicts have not learnt that simple lesson of how to stop cycles of vengeance; partly because leaders gain from maintaining the conflicts.

Compassionate motives were part of the United Kingdom efforts to rebuild a better world and get economies moving. So, from 1946 the United Kingdom built a National Health Service spearheaded by the passions of *Aneurin Bevan*, who argued that no society can be called civilized if sick people cannot get medical care because they did not have the money to buy it. It was a major disappointment that led to his resignation when the Atlee government later started to introduce prescription charges on certain items and funneling money toward rearmament. Once again, fear-based tribal psychology began to soak up resources. Nonetheless, the United Kingdom expanded education and created a range of other services (rail, water and electricity) for the public good. Taxes were high especially on the wealthy. It was a phase where we focused *on caring and sharing*, at least to a degree, wanting to raise the opportunities, services and health support for all and make some effort at redistribution of wealth. Again, part of this was compassionate, but there was also a recognition that having a sick and uneducated population and poor transport services was not good for business.

It was not long before these benefits created wealth and wealth settled into expectation for more. The uneven accumulation of wealth, along with fears generated by inflation re-ignited a more self-focused and less welfare focused politics. Changes in the social discourse, peddled by an increasing right-wing media that wanted to invigorate competitive behavior and “the market” regardless of the “fallout for the losers,” refocused governments on what they should aspire to do (Gilligan, 2011; Sachs, 2012; MacLean, 2017). The theme became “less is best” and the promotion of enterprise by facilitating “controlling and holding” onto the fruits of one’s enterprise. The problems of sink or swim free market economies producing vast inequalities of resources in power, back room deals and stifling social welfare was neatly ignored (Bakan, 2012; Stiglitz, 2012; Murray and Frijters, 2017; Biglan, 2020; Becker et al., 2021). Threat focused right-wing discourses and narratives began to stimulate different motives in our minds, not to care and share, but *to try to hold and control* our own claim on resources (Wilkinson and Pickett, 2010). This was not just greed based but fear based. Moreover, as divisions between the haves and have nots widen, with a media increasingly selling the idea that *wealth creates happiness and freedom from anxiety*, competition and fear of being one of the “have nots” or more recently ‘have less’ grew. Companies that needed to sell their products and compete in the marketplace came up with increasingly slick advertising campaigns linking their products to status, happiness and security. Until very recently, humans have never been bombarded with such constant stimulants of the drive system, social comparison and the aspiration to have more. For these and other reasons appeals to gain more freedom and control over one’s earnings, seek personal advantage and greater comforts, take responsibility for oneself and opt out of responsibility for others, flourished.

Through multiple channels, then the social discourses focused on what we should aspire to, how to set our core *personal values* and sense of self-judgment and entitlements: and the message was clear – personal wealth. While poverty creates misery, and degrees of wealth can afford protections from the challenges of life, partly because how our drive and resource seeking systems work, people wanted to *control and then hold* on to more and more, a process labeled affluenza (James, 2008) and materialism (Kasser, 2016). Consequently, there was a gradual shift away from the post world war II interest in the common good, and a caring and sharing of our collective wealth, to a system that fits and advantages money markets, short-term opportunism, unregulated, self-focused competitiveness, narcissism, psychopathic tendencies, and secret deals between the elites to maintain privilege (Bakan, 2012; Sachs, 2012; Hanson and Baker, 2017; Murray and Frijters, 2017; Biglan, 2020). Alarmed by the spiraling costs of the National Health Service, the Thatcher government of the 1980s began to find ways to start to privatize “Health” to reduce government responsibility and taxation and make health a “profit offering business” (Gilmour, 1993). In America, the sentiments for care and share were gradually unpicked by the Reagan government (Sachs, 2012). In the United Kingdom, it was Thatcher who believed that society is a collection of individuals pursuing self-interest, not a process of caring and sharing to be nurtured in and of itself. As Gilmour (1993) noted:

Margaret Thatcher wanted, through her economic policies, to change the heart and soul of the nation. She did achieve a transformation, but not presumably the one she intended. Britain did not change to an enterprise society. The change was in sensibility. British society became coarser and more selfish. Attitudes were encouraged which would even have undermined the well-being of a much more prosperous society. (p. 340)

Lost in the Aladdin’s cave of material resources we became dazzled by the ownership of things (and not having or losing them) or what has been called materialism. The problem with materialism is that it is addictive for “want more” (James, 2008; Wang et al., 2020). Wealth actually creates mind states that are less compassion orientated not more (Van Kleef et al., 2008). This is always the big problem, that as resource availability increases so does self-focused competitive behavior (Piff et al., 2018). Kasser (2016) sums up some of the key findings about the problems of materialism in his important paper.

Substantial evidence shows that people who place a relatively high priority on materialistic values/goals consume more products and incur more debt, have lower-quality interpersonal relationships, act in more ecologically destructive ways, have adverse work and educational motivation, and report lower personal and physical well-being. Experimentally activating materialistic aims causes similar outcomes. (p. 498)

After a certain point wealth does not increase happiness. In their introduction to the World Happiness Report, Helliwell et al. (2012) state

As one key example, the world’s economic superpower, the United States, has achieved striking economic and technological progress over the past half century without gains in the self-reported happiness of the citizenry. Instead, uncertainties and anxieties are high, social and economic inequalities have widened considerably, social trust is in decline, and confidence in government is at an all-time low. Perhaps for these reasons, life satisfaction has remained nearly constant during decades of rising Gross National Product (GNP) per capita.

There is also considerable evidence that materialism and self-focus competitiveness has increased social narcissism (Twenge and Campbell, 2009) along with unhappiness, anxiety, depression, social distrust, fear of failing and loneliness (Becker et al., 2021). Curran and Hill (2019) conducted a major meta-analysis of 41,641 participants across 164 samples on the increasing pressures of perfectionism between 1989 and 2016. They found that the levels of self-orientated perfection (feeling one has to be perfect), socially prescribed perfection (thinking others want perfection from the self) and other-orientated perfection (wanting others to be perfect) along with expectations and sense of entitlement have increased over the study. They argue that what sits behind this is: (1) the emergence of neoliberalism and competitive individualism; (2) belief in meritocracy, that success is all down to effort and competitive striving; and (3) anxious and controlling parenting. The latter increases as the competitive pressures within a society increase. They also note the role schools play in supporting neoliberal and striving ideals linking status and wealth-seeking to personal values which causes trouble “…., because individuals cannot avoid being sorted, sifted, and ranked by schools, universities, and the workplace, neoliberal meritocracy places a strong need to strive, perform, and achieve at the center of modern life.” (p.413). At the same time, leaving aside peoples compassionate behaviors during the COVID crisis, western societies have also become less compassionate, not more (Trzeciak and Mazzarelli, 2019) and entertainment has become more violent and vengeful as ways to excite minds that are already over stimulated by threat (Gilbert, 2018).

The great challenge to humanity at the moment is to regulate the runaway self-focused competitiveness which often happens in environments of high resource opportunity. Unregulated competitiveness has generated societies of haves, have nots and have lots and our distribution of inequality is extreme and economically and socially harmful (Toynbee and Walker, 2009; Gilligan, 2011; Stiglitz, 2012; Wilson, 2019; Becker et al., 2021). Wilkinson and Pickett (2010) have written extensively on the relationship of income inequality with crime and mental health difficulties and founded the Equality Trust to promote awareness and ways of addressing these issues. It is estimated that just 25 billionaires own over 50% of the world’s wealth (Elliott, 2019; Mathews, 2019). Not only this, but some of the wealthiest individuals in the world use their wealth to run industries which are damaging our ecology (fossil fuels, the making and using of plastics) and for political advantage undermining democratic processes in their favour (Pilger, 2007; Toynbee and Walker, 2009; Gilligan, 2011; MacLean, 2017; Murray and Frijters, 2017; Hickle, 2018). It is now recognized that although it was the deregulation (that Roosevelt had warned against in the 1930s) and the callous self-interest of the banking system that partly caused the 2008 financial crash, it was the least-well off that shouldered the burden of both the immediate fallout and the reparations paid subsequently. Rather than taxing the wealthy, we had austerity. The unfairness of such measures was clearly articulated in the recent *United Nation’s Human Rights Office of the High Commissioner* report (2018). Tragically not that much has changed. What politicians call “tough decisions” are more commonly callous decisions aimed to protect the wealthy and elites, with little regard for the affect they have on poorer communities (Gilligan, 2011; Curl and Kearns, 2015; Torjesen, 2016; Tooze, 2019). Recently, Observer journalist Rupert Neate (2020) noted that among the richest people in the world their collective profits over the COVID crisis have probably risen to over a trillion dollars. He quotes Frank Clemente of Americans for Tax Fairness who says “Their pandemic profits are so immense that America’s billionaires could pay for a major COVID relief bill and still not lose a dime of their pre-virus riches. Their wealth growth is so great that they alone could provide a $3,000 stimulus payment to every man, woman and child in the country, and still be richer than they were nine months ago”. While these individuals will be paying taxes, there seems no movement by most wealthy countries to seek increased taxes or even ask for voluntary contributions!

Many commentators have recognized that it was the austerity measures and efforts at privatization of the last 10 years that left the British NHS in such a state of underfunding, barely able to cope with everyday demands, let alone the crisis we have now that added to the COVID-19 death rates (McCoy, 2020). Estimates of increased deaths from untreated cancers and other conditions are tragic. United Kingdom governments had received numerous warnings from simulations and medical advisers that the country was not able to cope with any pandemic that would inevitably arrive and yet they chose to do nothing (Hopkins, 2020). This highlights again that recent political shifts have not focused on caring and sharing, but on supporting individuals to *control and hold resource*s for personal use which they believe will enable people to make decisions that advantage all of us. The evidence for a long time however, has been that there is little trickle down and it is mostly gushing up (Sachs, 2012; Carney, 2020). It is not clear if trickle down was ever really believed or was more of a political justification for low tax rates (Toynbee and Walker, 2009; Gilligan, 2011). So with the passing of the years, Western societies have become more self-focused, narcissistic, demanding (Curran and Hill, 2019; Twenge et al., 2014), and less compassionate (Trzeciak and Mazzarelli, 2019).

### Can Compassion Help?

Against the political movements of the last few decades, the tragedies of COVID-19 have illuminated many ways in which humans can be extraordinarily compassionate. In countries throughout the world, medical and other people have risked their lives to save strangers and vast numbers of people have provided help individually and or created “mutual aid” networks and groups to volunteer to help others and test vaccines, all common compassionate behaviors. At the time of beginning to write this paper in April 2020, many in the media suggested that COVID-19 could prove to be the turning point for Western society – the moment when we recognize our interdependency and the need to turn away from our neo-liberal, competitive self-interest past, toward a future built on recognizing we are a species of short, vulnerable lives with common desires to be happy and avoid suffering; to create a world rooted in our capacities for cooperation, caring and sharing (Sachs, 2012; Wilson, 2019; Carney, 2020). Writing in the Lancet, Professor of Public Health, Galea (2020) argues that compassion should be the focus for a future healthcare system which values our interdependency and desire to create good and effective healthcare and social justice for all, a sentiment many agree with (Gilbert, 2009; Sachs, 2012; Ricard, 2015; Ekman and Ekman, 2017).

If we are to begin to create a world that moves toward the common good, it will help to understand the processes that are underpinning these economic and political maneuvres (Biglan, 2020; Sachs, 2012; Wilson, 2019; Carney, 2020). There are powerful *evolved strategies* we need to be aware of if we are to create social contexts that support caring and sharing rather than unregulated, self-focused, competitive, controlling and holding resources.

### What to Do?

One of the key elements of compassion is to take a deep interest and look into the *causes* of suffering. Long term prevention cannot be addressed without understanding causes. One of the greatest dangers and illusions is to believe that political pursuits and values are somehow based on basic beliefs or are rationally chosen, when in reality, they emerge from conscious and unconscious sensitivity to threat and opportunity and the phenotypic activation of phylogenetically old strategies that pattern the brain. If it were a matter of basic beliefs, then providing people with clear evidence that caring and sharing will create a better world for all of us, change would be easy. What this paper has sought to do is to indicate processes that can be traced back to the evolution of resource competition strategies, and how different social and cultural contexts regulate these strategies. Neoliberalism has produced contexts that create minds and brains that are not well suited to the challenges of humanity, that needs urgently to move toward caring and sharing and intensify cooperative communities, nationally and internationally (Wilson, 2019). We have seriously allowed freedom to *control and hold* at the expense of social responsibility and indeed any form of responsibility including for our environment (Perry et al., 2013). What COVID-19 has illuminated is the extraordinary importance of context and focus in enabling different (i.e., invigorated caring) strategies to flow through communities. We are still basically hunter-gatherers who have a great interest in caring and sharing and are biologically capable of the most extraordinary acts of courageous and wise compassion, but also these can be easily turned off.

Hence, the most serious issue for humanity *is what is the fairest way for the distribution and use of wealth that is created everyday by the hundreds of billions of labor hours of human effort.* In his insightful evolution informed work on how to create cooperative communities and societies, Wilson (2019) echoes Boehm (1999) above:

One conclusion is that for any group to function as a corporate unit, the potential for disruptive self-serving behaviors within the group must be suppressed. This conclusion is so basic that it applies … to groups of any size. It is remarkable that the core problem confronting America in the early 20th century and today is the same problem that confronted the tiny Jamestown colony in the early 1600s: an extractive social organization that allows some to gain at the expense of others and the group as a whole. This is the human society equivalent of cancer. (p. 193)

Wilson also points out the well-known fact that every system in the universe is regulated and regulation itself needs to be regulated, because too tight and it constricts the process of change, too loose and it produces chaos. For those who seek deregulation, Wilson (2019) notes – “show me a deregulated organism and I will show you a dead one” (p. 193). In fact, many major economists now recognize that the crucial problems with capitalism are not so much competition but for what and how we can compete and what we do with the products of competition. The lack of regulation of competitive self-interest and at times pure callousness that values finance rather than human needs requires urgent attention (Stiglitz, 2012; Carney, 2020).

Constricting the power of others to ‘control and hold’ is only part of the solution because long term we want to create the conditions in our families, schools, economic pursuits and environments to feel safe, flourish and take an interest in each other – *to actually change the phenotypic profiles of humanity.* We are only going to do that if we create the cultures, including the childcare and education and resource distribution strategies accordingly. If we look at humanity as a whole, there are billions of brains in the world right now who could become wonderful doctors, scientists, composers and world changers who will never know they had such latent talents. They will not get a chance to develop their potential because they are locked into poverty and lack of opportunity. Many desire to find new ways of generating compassionate, cooperating communities, schools and nations, international sciences and businesses, working together to pool their knowledge, mutually supporting each other (Abel and Clarke, 2020). To do this however, we must not be naive about the evolved nature of the human mind because unless we recognize and engage with the serious inhibitors of caring, sharing and compassion, and the ease of shifting into callousness even cruelty, we will fail (Gilbert and Mascaro, 2017).

We also need urgent changes in how we govern ourselves at local and international levels. Many political theorists and commentators recognize that western political systems have run their course and not fit for purpose. We have voting systems rather than true democracies. In many elections, few politicians get endorsed by a majority of eligible voters, and in multi-party states, many will have more voting against them than for them, hence why many people opt out of participating. In addition, people are often asked to make decisions on complex questions without any depth of insight. Policies require complex knowledge. When the British people were asked to vote on leaving the European Union few understood what a customs union actually was and instead some voted on more media fueled fears of immigration and issues of sovereignty. We have created societies that actually are increasingly complex and technical. For this and other reasons, individuals are looking toward different ways of engaging “democracies” such as the concept of *crowdocracy* (Watkins et al., 2016). Watkins et al. noted that there are many vested, powerful interests in maintaining the *status quo* that we do not know how to address. They also argue that many of our problems are what they call ‘wicked problems’ in the sense that they are multi-factorial, multi-causal and the solutions are going to be multidimensional. Not so easy.

Many have suggested taking the business financing of politics out of the game as a start. In addition although collaborative work happens behind the scenes in the select committees and at other places it is obvious that competitive politics has degenerated, where the main purpose of debate is to ridicule the other, stirred on by the media who like to sell the blood on the carpet. To paraphrase consider how common it is to hear the media talking about the need for a leader who could “damage and land blows on the opposition.” Commentators have sometimes referred to the political debates between leaders as “no one was able to land the knockout blow.” At times politicians seem unaware of the kinds of minds that are being stimulated in their interactions with each other. The moment a politician comes on the television or the radio, you know exactly what they will start off with, which is an attack on the other side and where possible make them appear incompetent, immoral or dangerous. This not only drives diverseness but as recent events with the Trump administration shows is increasingly dangerous. This is not a psychology that the world needs.

Indeed, it is very hard to be aware of how much our minds are puppets to the social discourses we are embedded in. From families to cults to social groups, religious or political movements, it is not uncommon for people to need to get out of a toxic environment before they are able to reflect on how the environment controlled ‘their minds’. One of the key proposals should therefore be that politicians need far more psychological support and opportunities to stand back, understand and reflect on these processes and ideally agree to build cultures that bring out the best of discussion, debate and decision-making. As new president Joe Biden has recently highlighted it is possible to disagree without being disagreeable; to be respectful rather than ridiculing; to promote cooperation rather than a winner takes all.

The fact is that left and right are now old tribal labels of little value. In reality we are having to manage two profoundly powerful resource regulation strategies that are operating at *phenotypic levels*. Politics is not just about the management of values. It is the management of brains and bodies too; and in particular the dark side of humanity. There is now considerable evidence that when we feel safe we are biologically different to when we feel threatened (Slavich, 2020). It is important then to recognize that capitalism actually generates high stress and threat because of the continued process of change, of jobs appearing and disappearing and the fear of losing is partly what drives it; you have to be better than your competitors or you don’t get the job (Becker et al., 2021). Whether picked up in childhood or from culture, threat drives right wing control and hold strategies.

Another problem is that in our ancestral groups our drive and resource seeking motives were regulated by resource shortage and their immediate use. There is no natural regulator now for resource seeking drive motives. The desire for “more and better” is partly what made us the species we are with our advanced science but unregulated it is addictive, destructive and turns everything and everyone into a resource; it drives the criminal mind as much as it drives to the ruthlessly ambitious. Wanting more and better is of course a natural psychology and the psychology of contentment and sufficiency is tricky, particularly when people socially compared to their own reference group (Naish, 2008; Cordaro et al., 2016).

Nonetheless there are many things we can do to promote caring and sharing and offset affluenza! Hence there are some obvious, commonly suggested, steps to explore (Ryan, 2019; Wilson, 2019; Abel and Clarke, 2020). What is exciting and inspiring is that there is an increasing bubbling cauldron of many fascinating and exciting ideas that are emerging on how to create a better world. Researchers are looking at the complex interactions between leaders and voters that stimulate fear and divisiveness because it is a two-way process of some challenge (Mols and Jetten, 2020). Politicians are looking at how to reform political systems, because competitive politics is obviously failing us and attracting the wrong type of minds. We need minds to address the challenges of cooperative problem solving. Businesses are just beginning to engage with the moral dimension of their profession and recognize that they should not support colleagues who create businesses that are harmful to the ecology, exploit workers and hide taxes and where profit is the only judgment of success. There are increasing calls for “moral business” as internet searches will reveal. All of us then can play a role in changing the world for our grandchildren.

#### The Individual

• We are desperately in need of better psychoeducation on the nature of the human mind, in particular, its ease of turning to the “dark side,” and that we have brains and minds built for us by our genes and social contexts. Much, of what we become and what we believe in is not chosen in the first instance, but are products of an evolved brain choreographed by its social environment. It is a serious narcissistic blow to realize that we are a short-lived, evolved entity etching out its hopes, fantasies and desires according to its programming. This was Siddhartha’s insight on his road to Buddhahood enlightenment, although of course, he used different concepts and language. Over 2000 years ago the contemplative traditions, particularly but not only Buddhism highlighted the fact that we are different from other animals because we have competencies to be mindful, to become an observer of our own mind and mind aware. This gives us the potential to “wake up” to the ways in which we can be regulated by passions and desires in unhelpful and destructive ways. Compassion training is a way of retraining and reprogramming our minds. If on our short stay in this life, we want to be more than a puppet to the above strategies that flow through us, then this requires us to gain insight into our programming, learning mindful awareness and what we can do to facilitate more compassion focused states of being. Hence, mind awareness, mindfulness and compassion training need to be textured in our education system, from schools to universities, businesses and work places (Ricard, 2015; Coles and Gent, 2020). The classic film, the Matrix and science fiction series Westworld played with this idea; “waking up” is not necessarily an easy solution, but one with huge dividends (Ricard, 2015).
• Caring and compassion are potential strategies, wired into us with their own physiological architecture (Keltner et al., 2014; Mayseless, 2016; Carter et al., 2017). Hence, we need clearer and invigorated psychoeducation about how a compassionate orientation to self and others has profoundly important impacts on the brain and physiology including the autonomic nervous system, cardiovascular system, immune system and various frontal cortical circuits that regulate emotion (Seppälä et al., 2017). It promotes ethics, but also physical and mental health (Brown and Brown, 2015, 2017). Our research on how to train our brains to stimulate compassion systems is improving all the time and these trainings need to be made increasingly available in schools, businesses and other places. Compassion is not just about soothing, but it is also about energizing for courageous and wise action.
• While compassion is about sensitivity to suffering, it is also about the prevention of suffering. Compassion is based on a sense of personal and collective responsibility (as in COVID-19). The basis of altruism is that we are prepared to make sacrifices for the benefit of others and when we do that, the overall effect turns out to be beneficial for all of us (Ricard, 2015).
• Around the world, there has been an explosion of interest in mind training. Mindfulness and compassion are the main focus with increasing evidence of their person benefits. The degree to which such practices will indeed stimulate a will to see care and share strategies permeate their cultures is unclear. It is well known that there are many narcissistic business leaders who adopted mindfulness, but had not really engaged compassion. Unless compassion motivation becomes the intention behind mindfulness, there is no guarantee that it will produce movement toward the common good.

#### Social

• Although lacking an evolutionary lens, many political philosophers, psychologists, sociologists anthropologists, and other commentators recognize that democracies, as currently formed, are unhelpfully competitive focusing on the undermining, shaming and suppression of competitors not the promotion of the best (Mulgan, 2006; Sachs, 2012; MacLean, 2017). Many recognize the need for socially sensitive politics with compassionately sensitive individuals that seek to create social contexts and narratives that focus on desires to *live to be helpful, not harmful*. COVID-19 has shown that this is possible, and is desired by many, but it needs to be harnessed. This compassion motto *of live to be helpful, not harmful* can be taken up by business, politics and many other processes (see the “compassion in politics’ movement”). Imagine if this motive, not just the profit motive, was adopted by shareholders and businesses alike. Wouldn’t it be amazing if organizations like Confederation of British Industry adopted that motto for its members as the equivalent of the Hippocratic Oath: *that all businesses will seek to contribute to humanity not to harm it*. In fact, my experience of working with some of the younger business people is that this is exactly what many want to bring into the world, while recognizing that there are also many in business who would oppose it because they want to continue with their plastic and fossil fuels productions, deforestations, and stock market gambling to make money regardless of long term harm. Indeed, COVID-19 revealed how callous some very wealthy business people were in how they supported their workers or used the situation to profit.^1^ Care-focused social mentalities just seem to be “unavailable”; the algorithm does not seem to be working for these individuals. But, there are also many business individuals who really stepped up to the mark. Indeed, putting “moral or ethical business” into a search engine reveals some truly inspiring efforts at compassionate and ethical business. If talented entrepreneurial minds make compassion, appropriate cooperativeness, fairness, respectfulness and ethics centre to their mission, not *just* profit, the future could be rosy, especially if they insist on the media and politics provide them the contexts to do it (Carney, 2020). There are many potential innovative ways of bringing a more caring and sharing orientation to the world; we just have to put our minds to it.
• Repeatedly, we have understood that we need to develop forms of social, economic and political regulation, and this includes having monitoring systems not only for preventing criminality, but also the distorting aspects of resource accumulation in the hands of the few. This will include careful monitoring of the ecological harm some companies do and the importance of appropriate taxation. There are many different types of taxation, such as the Tobin tax, carbon tax that need to be revisited. We need to recognize that through the lens of a hunter-gatherer mind, individuals who accumulate vast wealth are being allowed to cheat. It is only natural that we want the best for ourselves, not the worst. So, it is important not to confuse the desire for the comforts of life (I love mine), with the issue of the regulation of resource distribution and the pursuit of wealth. Humans will always compete and regulated competition can be very helpful; unregulated it becomes harmful.
• There are increasing movements, such as the movement to develop compassion in politics^2^ with increasing (and always have been) younger politicians, very committed to trying to regulate conflicts of interest to produce a more caring and sharing culture, and recognize that the aggressive rivalry and competitiveness of politics are harmful.
• Moreover, that political discourse is overly influenced by a media who are interested in stimulating the emotions of conflict (Mulgan, 2006; Sachs, 2012; MacLean, 2017). Changing the tone of the media is essential (Sachs, 2012). The media could stop peddling the fantasy that they report the news, they don’t make it. In fact, there is considerable evidence that the media can shape our emotional reactions to the events around us for better or for worse. Reporting ‘what sells papers’ is not the same as reporting objectively and with the desire to avoid stimulating harmful divisiveness. Important too is reporting on the good in the world and on hope. Again there is no simple view or solution but it starts with the *desire and motive to try* to create a media that will build towards a fairer, more compassionate, caring and sharing world. This can start from the inside but also what we buy and attend to. This is urgent now with the multiple information flows and the peddling of false news, conspiracies, fears and divisions.
• While this paper has focused on the inhibitors of compassion, we also need to celebrate and take hope from the fact that there are many wonderful facilitators. Today we see more open discussion of the problems we face. There are entrepreneurs working to improve farming and soil quality, alternatives to meat, alternatives to plastics, find ways to create more green energies and replace fossil fuels for aircraft. There are exciting reforesting projects and how to get the plastics out the sea. Recent changes in online training has opened many areas of the world that would previously have been excluded by distance. And of course, medical science continues to astound. While pushing for efficiencies can be heartless and cold, building cheaper and faster can also be a source of great benefit to all. In the prevention of suffering compassion seeks to build on the good.
• Create more opportunities for people to be contributors to their community, which may require government action to facilitate compassionate community care programs, for example, supporting sports programs, music or literary skills in poor communities. Support policing programmes of community befriending, because compassionate policing is an essential part of a compassionate society. As we see throughout the world today, police can easily be turned against the population and become oppressors. Governments can facilitate street parties to celebrate the ending of the virus for example and do that every year. Create visions of a caring and sharing within the international community, recognizing that this will have a major impact not only on values, but also on brains and even our epigenetics over the long term. However, as the history of the United Nations show despite high ideals, good intentions and some success this is going to be no easy task because of the enormous inhibitors and desires for control and hold at the level of leaders and countries that we are up against.

Finally, we need to find ways to promote individuals into positions of power who are bright, but also show they have the talents for empathic co-operation because a world run by narcissistic, competitive leaders with ‘dark triad personality traits’ has been, and will continue to be a disaster. In other words, we should not underestimate the dynamics of, and regulation of, those who have access to power (Gilligan, 2011; Owen, 2020). This is actually quite tricky and needs considerable thought and research because they know how to present their ‘caring and protection’ credentials to attract followers. Yet the evidence is that when we have prosocial leaders our social contexts can change for the good, whereas when we have anti-social leaders (Basran et al., 2019) we enter the dark realms of competition, tribalism and division. Gilligan (2011) makes the point that even when people know a leader is dangerous and unfit for office, there are few ways they can be removed on that basis. From Hitler to Stalin (and others today), it was very obvious to many that these individual were callous, ruthless and dangerous and would do great harm—but there was, and still are, few mechanisms to stop them from gaining power. Indeed, others around them can be complicit and feed off their ruthlessness for personal gain themselves.

## Conclusion

This paper began by highlighting the realities of life; that all biological life forms are vulnerable to injury, decay and die. Human suffering is greater, because we know that we suffer, we know that we die, we know we can be in pain, we know that everything is impermanent and in a constant state of flux and change. As individuals, as societies and as a species, we need to seriously consider how we address the reality that biological life is about suffering and how this challenges us. A civilized society must first and foremost seek to address suffering and not cause it. Caring and compassion are the principle motives by which to address suffering, but it is very easy for us to dissociate or be in denial of suffering, distracted by the modern comforts and the threats of economic loss and inferiority.

Evolution has created both many forms of suffering, but also potential solutions. At the root of evolution is the management of conflict. The idea that the only way to engage in conflict is to create “nature red in tooth and claw” or the sink or swim fates of unregulated markets is wrong, because conflict can be dealt with cooperatively and with sharing (Wilson, 2019). However, Van Kleef et al. (2008) and the extensive work of people like Paul Piff (Piff et al., 2018) have shown that as people become wealthier, they become less compassionate. This is because in competitive (in contrast to sharing) environments, the control and hold strategies can become more “adaptive” and influence the psychophysiological organization of the mind. As indicated all the way through, this is related to how our very ancient, evolved gene-built, mental algorithms and programming interacts with the modern world creating particular kinds of brains and phenotypes. As part of this, there is the need to understand the *inhibitors* of cooperative and caring and sharing behavior (Gilbert and Mascaro, 2017). There is an old saying from a galaxy long ago and far away “beware the power of the dark side.”

We increasingly understand more about the human mind than at any time in history, including our genes, evolutionary history, and the emergence of different phenotypes in different cultures. Crucially too research is revealing more and more ways in which compassion impacts our brains, bodies and communities. This is gradually filtering through into education and into businesses, which is inspiring. However, if we do nothing about these ancient *control and hold* resource distribution strategies, that since the advent of agriculture has wreaked havoc in the world, then unregulated we run into the well-known risks that as the wealth of the world increases, our ecologies will become increasingly poisoned and stripped bare, the divisions and disparities of wealth will get worse, empathic concern for the less well-off will drift away into populist movements, conflicts will intensify and as today rage will increasingly replace argument. The arms industry will blindly strip vast resources to fuel research and the manufacture of new weapons and artificial intelligent robot soldiers likely to be sold to narcissistic leaders to suppress their own population. Narcissistic leaders with bombs in their pockets, are not focused on peaceful forms of coexistence, but the need to build ever great defenses and present themselves as protectors. Groups will continually fracture into subgroups, possibly populist ideals of self and group importance and entitlements or around religions that offer protection and a source of specialness and well defined group identity. Some will become terrorist groups because damaged narcissistic leaders will spread fear in the group. The media that trades on conflict and tragedies will get worse. The backroom deals of the elites will get tighter, become more secretive and seek to cheat on their responsibilities. We would be heading for Blade Runner worlds.

At the end of the day, the solutions to all this has to be informed by science; to understand the facilitators and inhibitors of what we desperately need i.e., cooperation, care and compassion (Ricard, 2015; Gilbert, 2018; Wilson, 2019). We can use science for research and the generation of new ideas to find new forms of sharing and caring that reaches humanity as a whole; keeping in mind that this was not the evolutionary context for caring and sharing. Evolution has handed us a mind that is capable of great compassion, but also in the context of large groups and storable and accumulable wealth, absolute demonic terror. Whether we follow spiritual beliefs or not, that is the battle that goes on in our individual minds, in our communities and our societies. It is not a battle between good and evil, it is a battle between two resource distribution strategies that are millions of years old. As we come to understand how we are programmed (against our awareness) and how our minds are emotionally and motivationally orchestrated by these strategies, we may decide enough is enough. We have a degree of intelligence and mindfulness and can decide to take control of the steering wheel. Compassion turns out to be the most courageous and wise of all of our motives.

## Author Contributions

The author confirms being the sole contributor of this work and has approved it for publication.

## Conflict of Interest

The author declares that the research was conducted in the absence of any commercial or financial relationships that could be construed as a potential conflict of interest.

## Footnotes

1 https://www.veracitysearch.co.uk/insights/2020/3/4/coronavirus-compassion-or-callousness

2 www.compassioninpolitics.org
